# Impact of chronic low-dose external gamma- and internal tritium beta-irradiation on the gut microbiome in the context of intestinal tumorigenesis in *Apc^Min/+^* mice

**DOI:** 10.1128/msystems.01156-25

**Published:** 2026-03-17

**Authors:** Holly Laakso, Manar Hashem Taha, Matthew Flegal, Joel Surette, Mohamed Mysara, Dmitry Klokov

**Affiliations:** 1 Isotopes, Radiobiology, and Environment Directorate, Canadian Nuclear Laboratories 417642, Chalk River, Ontario, Canada; 2 Bioinformatics Group, Center for Informatics Science, School of Information Technology and Computer Science, Nile University 120639, Sheikh Zayed City, Egypt; 3 Microbial Biotechnology Research Unit, Nuclear Medical Applications, Belgian Nuclear Research Center (SCK CEN) 74873 , Mol, Belgium; 4 Laboratory of Experimental Radiotoxicology and Radiobiology, Department of Health Research and Expert Assessment, The French Authority on Nuclear Safety and Radioprotection (ASNR) 52834, Fontenay-aux-Roses, France; 5 Department of Biochemistry, Microbiology and Immunology, University of Ottawa 6363, Ottawa, Ontario, Canada; Technion Israel Institute of Technology, Haifa, Israel

**Keywords:** low-dose radiation, gut microbiome, tritium, gamma radiation, colon cancer, *Apc^Min/+^*

## Abstract

The gut microbiome (GM) plays an essential role in health, and its dysbiosis can increase the risk of colon cancer. While the detrimental effects of high-dose ionizing radiation on GM have been documented, little is known about the effects of low doses, including from internal exposure to tritium, which is produced by nuclear power generation and emits beta radiation, making it a public concern. We examined the effects of chronic irradiation with internal tritium beta radiation or external ^60^Co gamma radiation on GM and intestinal tumorigenesis in the *Apc^Min/+^* mouse model of colorectal cancer. Mice were exposed to tritiated drinking water (HTO) or gamma radiation at cumulative doses of 0, 10, 100, and 2,000 mGy, followed by intestine, blood plasma, and fecal sample collections at 12, 16, and 20 weeks of age. HTO- and gamma-exposed cohorts had distinct tumor size and multiplicity patterns, with non-monotonous dose-responses. Complex patterns of blood cytokine changes with age, dose, and type of irradiation were recorded. GM analyses using 16S rRNA amplicon sequencing revealed significant changes in alpha and beta diversity in irradiated mice compared to controls, indicating altered microbial dynamics. HTO and gamma radiation induced distinct microbiome changes that did not correlate with tumor and blood cytokine readouts. Our results suggest that chronic exposure to low-dose gamma- or internal HTO beta radiation can affect GM in a radiation type and dose-dependent non-linear manner. Our results provide novel insight into the effects of low-dose gamma- and tritium beta radiation on GM and a possible association with tumorigenesis.

**IMPORTANCE:**

Low-dose ionizing radiation is one of the few environmental stressors that simultaneously reshapes host physiology and the structure-function landscape of resident microbiomes, yet mechanistic insight at ecologically relevant doses has been scarce. By integrating longitudinal 16S rRNA profiling, multiplex cytokine analyses, and quantitative tumor phenotyping in the *Apc*^*Min/+*^ mouse model, our study demonstrates that continuous exposure to either external ^60^Co γ-photons or tritium beta particles perturbs gut microbial community structure in radiation-quality-specific ways and that these shifts track with, and sometimes precede, complex, non-monotonic changes in intestinal tumor burden. The work expands the traditional radiobiology focus from host-centric DNA damage to a systems-level view in which microbe-host-radiation interactions form a dynamic network influencing early colorectal carcinogenesis.

## INTRODUCTION

The gut microbiome (GM), a complex ecosystem of microorganisms in the gastrointestinal tract, plays a pivotal role in human health by influencing metabolic and physiological processes (1). Dysbiosis, or microbial imbalance, has been linked to a range of health conditions in humans and animals, including depression (2), deterioration of cognition (3), obesity (4), type 2 diabetes (5), immune response to cancer and radiotherapy (6), various cancers (7–10), including colorectal cancer (CRC) (11). The colon hosts the highest concentration of microbes in the intestinal tract, so it is not surprising that extensive evidence supports the association between the GM and the initiation, promotion, and progression of CRC (12, 13), as well as its metastasis (14).

CRC represents a major global health burden, leading to around 940,000 deaths globally in 2020 and ranking as the second most common cause of cancer-related deaths. Each year, approximately 1.93 million new CRC cases are diagnosed, with a rising incidence especially in developed nations and among people younger than 50 years old (15–17). Exposure to ionizing radiation (IR) in cancer patients has been recognized as one of the risk factors for secondary CRC (18, 19). The association between IR and CRC is further evidenced by the Life Span Study of atomic bomb survivors in Japan, which demonstrates a dose-response relationship, indicating higher CRC risk with increased radiation dose (20). Nowadays, exposure to low doses of ionizing radiation (LDR) affects much larger human cohorts in various scenarios, including medical diagnostic imaging (acute LDR) and occupational settings (chronic LDR), highlighting the critical need to understand how even low levels of radiation can influence CRC risks and whether those could be mediated by the altered GM.

Exposure to IR can lead to alterations in the composition, richness, and diversity of the GM, both in animal model studies (21) and in humans (22). These alterations are characterized by the promotion or suppression of specific microbial species, which consequently alter their gene expression and functional capacities; for instance, induction of oxidative stress and activation of pro-inflammatory cytokine signaling (e.g., interleukin-6 [IL-6] and tumor necrosis factor-α [TNF-α]), production of genotoxins such as colibactin and hydrogen sulfide, and modulation of immune responses, including Treg/Th17 imbalance—all of which contribute to a microenvironment conducive to intestinal tumorigenesis (17, 23). IR-induced dysbiosis has been associated with a range of changes, including an increase in the abundance of pathogenic bacteria such as Proteobacteria and Fusobacteria, and a decrease in beneficial bacteria like Faecalibacterium and Bifidobacterium (22). Most frequently, this IR-induced dysbiosis has been observed at high radiation doses in cancer patients undergoing radiotherapy (24). Remarkably, the GM plays a vital role in modulating the effects of IR on the host, with specific bacterial taxa like Lachnospiraceae and Enterococcaceae being associated with the restoration of hematopoiesis and gastrointestinal repair after exposure to high doses of IR (25). However, whether LDR can cause similar effects on the GM remains unknown. Only 2 out of 19 mouse studies on the effects of IR on the GM reviewed by Fernandes et al. (21) used low doses (≤100 mGy as defined by the UNSCEAR [26]), demonstrating mild or no effect on the GM composition and diversity/richness at low doses. Wild species of bank voles (*Myodes glareolus*) living in highly contaminated areas in Chernobyl, Ukraine (and thus exposed chronically to internal and external IR), had alterations in the GM bacterial ratios, but no significant differences in overall bacterial richness, compared to the microbiome of bank voles located in low- or uncontaminated sites (27).

Among the types of IR humans are exposed to, internalized tritium—a beta particle emitting radioactive isotope of hydrogen—warrants special attention. This is primarily due to its anthropogenic generation and release into the environment, either as a byproduct of nuclear power generation or as a result of accidents like the Fukushima disaster (28). The Japanese government’s decision to release wastewater from the Fukushima accident containing large quantities of tritium into the ocean starting in 2023, under the ALPS project, has caused significant societal concern and inter-governmental unease (28). These concerns stem from the potential impact of tritium on environmental and human health (29, 30). Despite long-standing investigations into tritium’s biological effects (31), significant inconsistencies in evaluating its health impacts remain, resulting in a wide range of values for its relative biological effectiveness, a critical factor in radioprotection risk assessment (32). Consequently, regulatory levels of tritium in drinking water vary significantly among countries, reflecting these uncertainties (30). With the renewed interest in nuclear power as a sustainable energy source and the prospects of nuclear fusion—which involves the use of tritium (33)—it is crucial to understand the mechanisms of LDR-induced health outcomes, including CRC.

In this study, we test our central hypothesis that chronic LDR, as either internal beta radiation from ingested tritium or external gamma radiation, can cause significant changes in the mouse microbiome in the context of CRC using the *Apc^Min/+^* mouse model. Furthermore, as the mouse ages and tumorigenesis progresses in the intestines, the microbiome can continue to alter in response to the immune and tumor microenvironment. Thus, this study allows correlation of dysbiosis to intestinal tumorigenesis and allows comparisons between the effects of internal beta particles and external gamma photons on the GM.

## MATERIALS AND METHODS

### Animal protocol summary

C57BL/6J-*Apc^Min/+^* (*Apc^Min/+^*) male mice and C57BL/6J (wild type [WT]) female mice were purchased through Jackson Laboratories (Bar Harbor, ME, USA). Mice were bred and housed in a specific pathogen-free Biological Research Facility (BRF) at Canadian Nuclear Laboratories (CNL; Chalk River, ON, Canada) following standard animal care protocols. Pups were born roughly 20 days after the start of breeding. At 3.5 weeks of age, pups were sexed and tattooed on tails for identification, and oral swabs were collected for genotyping and then shipped to the Genotyping Center of America (USA) for a PCR-based genotyping assay. Male mice were used in the study, with the *Apc^Min/+^* mice housed in separate cages from the WT. Mice were randomly distributed into cages and groups and housed individually in duplex ventilated cages (Thoren Caging Systems Inc., Hazleton, PA, USA) with automatic ventilation and watering and were provided Charles River Laboratories rodent chow (Frederick, MD, USA) *ad libitum*. For HTO-exposed groups, cages were placed on a negative-pressure rack to prevent any inadvertent tritium transfer to control animals. A constant temperature of 23°C and a 12-h light/dark cycle were maintained in the facility. For survival analyses, animal health was initially monitored at cage-change intervals (7 days) before the age of 8 weeks, and later, animals were monitored daily for signs of illness or suffering. Mice that showed clinical signs reaching humane-intervention points, as defined by the facility veterinarian, were euthanized. Tests for pathogens were performed routinely, and all tested mice were negative.

### Exposure to internal beta radiation via ingested HTO

For HTO exposures, tritiated water stock (3.7 × 10^9^ kBq/L) obtained locally from the National Research Universal reactor facility at Canadian Nuclear Laboratories was used. Tritiated stock water was diluted in reverse osmosis animal drinking water. *Apc^Min/+^* mice were exposed at 4 weeks of age for 56 days to continuous ingestion of 4.53 MBq/L HTO (equivalent cumulative dose 10 mGy), 45.3 MBq/L HTO (100 mGy), 906 MBq/L HTO (2,000 mGy), or sham treated (0 mGy). The final concentration of tritium in HTO water preparations was confirmed by liquid scintillation counting. HTO was provided continuously throughout the 56 days for mice to ingest *ad libitum* and was replaced with freshly prepared water every 2 weeks. Assuming a daily water consumption of 5 mL and a mean mouse body weight of 20 g during the experiment, this represents a mean ingestion of 1.13 kBq/kg of body weight/day (0.179 mGy/day), 11.3 kBq/kg of body weight/day (1.79 mGy/day), and 226.5 kBq/kg of body weight/day (35.8 mGy/day) (33). Approximately 10 mice were randomly assigned to one of these exposure groups or the sham-treated group. Control mice were maintained in a rack with positive air pressure to avoid tritium contamination through breathing air. Negative air pressure was maintained in the racks hosting mice exposed to HTO.

Kinetics and estimation of absorbed dose were extrapolated from previous work that demonstrated during drinking HTO ingestion that the concentration of tritium in C57BL/6 mice reached a peak after about 10 days in a 30-day exposure model and dropped rapidly after the cessation of HTO administration. The total amount of tritium absorbed by the soft tissues was determined via liquid scintillation counting. The maximum concentration of tritium reached was only about 50% of that in the water consumed. Assuming equivalent equilibrated uptake during the 56-day exposure:


(1) $$Xd=\frac{D\times \beta \times E\times T}{2}.$$

*Xd* is the dose to mouse in Gy per day; *D* is drinking water tritium concentration (Bq/L); β is the mean energy of emitted beta particles from tritium decay, 5.7 × 10^−3^ MeV; *E* is the conversion factor from MeV to Joules, 1.6021 × 10^−13^ J/MeV; *T* is the number of seconds in one day (86,400 s); and the divisor of 2 accounts for 50% radionuclide uptake in soft tissue (34, 35)

The background dose rate in the animal room, where both tritium-exposed and sham control groups were housed, was approximately 0.06 µGy/h (1.44 µGy/day), measured using a Bicron Micro-Analyste Micro R Survey meter (Bicron Electronics Co., Canaan, CT, USA).

### Exposure to external gamma radiation

At 4 weeks of age, male *Apc^Min/+^* mice were exposed to an open beam ^60^Co source (Gamma Beam 150C; Nordion) in the 30-m-long Gamma Beam Irradiation Facility at Canadian Nuclear Laboratories. The Gamma Beam 150C had a total activity of approximately 5.46 Ci ^60^Co. Mice were irradiated in separate batches due to the disparity in dose rates between the low-dose (10 and 100 mGy) and the high-dose (2,000 mGy) groups. Both physical shielding (1.75 inches of lead shielding) and the distance of the animal cages from the primary source allowed the required dose rates of 7.7 µGy/h (for the total absorbed dose of 10 mGy) and 77 µGy/h (100 mGy). The high-dose cohort received an unshielded dose of 1.49 mGy/h (2,000 mGy). Dosimetry was completed using Exradin A8 ion chamber and SuperMax electrometer readings to determine average gamma radiation dose rates in the irradiation hall. During irradiation, dosimetry measurements were collected using passive dosimeters containing Harshaw thermoluminescent dosimeter TLD-100e LiF:Mg, Ti chips (Harshaw Chemical Co., Solon, OH, USA) placed in empty mouse cages distributed at the corners and centers of each animal rack to record total absorbed gamma dose. Average dose rates for gamma radiation were derived by dividing the total gamma dose, determined by the mean TLD measurement per animal rack, by the total exposure period. Average dose rates were adjusted for beam downtime that was necessary to accommodate animal husbandry activities. While in the gamma beam hall, mice were housed in wooden racks in Thoren cages at 23°C–24°C, 37%–40% humidity, and an atmospheric pressure of 754–759 mmHg.

### Euthanasia and sample collection

At predetermined time points (4, 12, 16, and 20 weeks of age), mice were weighed and anesthetized by isoflurane inhalation followed by cardiac exsanguination and euthanasia by cervical dislocation. A variety of tissues and samples were collected for subsequent analyses. Intestines were extracted, cleared of the mesenteric tissue, and flushed with cold PBS. For intestinal tissues, Swiss rolls were made of the entire uncut tissue (small and large intestine, and colon) inside a histology cassette and submerged in 10% formalin for 24 h.

### Blood test and multiplex cytokine analysis

Peripheral blood was collected by intracardiac puncture into a heparinized syringe. To collect plasma, an aliquot of peripheral blood was centrifuged at 2,000 × *g* for 10 min at 4°C. Plasma was carefully transferred into 1.5 mL Eppendorf tubes, snap frozen in liquid nitrogen, and stored at −80°C. Blood plasma samples stored at −80°C were thawed on ice, and aliquots of 50 μL were collected for analyses. Concentration of select cytokines, chemokines, and growth factors in the plasma was analyzed using the Bio-Plex 200 system and Bio-Plex ProTM Mouse Cytokine 23-plex panel, Group I (Bio-Rad, Hercules, CA, USA). Each plate was designed and assembled according to the manufacturer’s instructions. Each plate was run twice on the Bio-Plex 200 array reader, using both the low and high photomultiplier settings with the appropriate standard selected. Data were analyzed using the Bio-Plex ManagerTM 6.1 software.

### Histology, H&E staining, and tumor scoring

Upon extraction, intestinal Swiss rolls were fixed in formalin for 24 h. Tissues were then transferred into 50% ethanol (30 min), then 70% ethanol, and kept for no longer than 3 days. Tissues were processed using an automatic tissue processor (Thermo Scientific) followed by embedding in wax/paraffin blocks using a tissue embedder station (Leica Biosystems). The resulting wax blocks were stored at room temperature until sectioning. Using a microtome (Leica Biosystems), tissue blocks were cut into 4 µm thick sections, with 8–150 µm between sections, until the Swiss roll was cut. This resulted in 8–16 sections per Swiss roll. Sections were placed on clean microscope slides and stored at room temperature for up to 4 days prior to performing hematoxylin and eosin (H&E) staining. H&E staining was performed using an autostainer (Leica Biosystems). H&E-stained slides were immediately mounted with coverslips using mounting medium (Permount, Fisher Chemicals). Once the mounting medium solidified, the slides were stored at room temperature until imaging. H&E slides were imaged using an automated slide scanner (Leica Biosystems), with sections imaged at 1.5× and 10× magnifications. Blindly encoded digital images of the slides were transferred to a trained histopathologist at the University of Ottawa, Ottawa, ON, for analysis and scoring. On each section, the number and length of adenomas were measured, followed by the integration of the data from all sections per mouse to eliminate the data from the same adenomas. This resulted in a total score of tumors per mouse, with the maximal size of each adenoma recorded. The tumor metrics were further refined into the number of small (<500 µm), intermediate (500–1,500 µm), and large (>1,500 µm) tumors for a better understanding of tumor growth dynamics. For the comparison of tumor length and counts between groups, the Mann-Whitney *U* test was used, with *P* ≤ 0.05 considered significant.

### Collection of fecal samples from the *Apc^Min/+^* mice

At the time of euthanasia, any defecation (fresh fecal pellets; one per mouse) was taken using sterile forceps, placed in a 1.5 mL Eppendorf tube, and flash frozen in liquid nitrogen. If the mouse did not defecate at the time of euthanasia, the rectum feces (one per mouse) were removed at dissection <10 min following morbidity, placed in a 1.5 mL Eppendorf tube, and flash frozen. All samples were then stored in a −80°C freezer until DNA extraction.

### DNA extraction from fecal samples

DNA samples were extracted using ZymoBIOMICS DNA Microprep Kit (Zymo Research) according to the manufacturer’s instructions. Briefly, fecal samples were placed on ice and weighed prior to extractions. Weights generally ranged from 10 to 65 mg, largely dependent on the age of the mouse and severity of intestinal tumorigenesis at the time of euthanasia. Immediately prior to lysis, 20 μL of ZymoBIOMICS Spike-in Control I (High Microbial Load) (Zymo Research) was added to each sample. This spike-in control contained equal cell numbers of bacteria, *Imtechella halotolerans* and *Allobacillus halotolerans,* and served as an *in situ* positive control for DNA-sequencing measurements. Samples were then lysed using the provided BashingBead tubes and lysis buffer with the TissueLyser II (Qiagen) set to 30 Hz for 5 min in cassettes chilled to −20°C.

Following lysis, samples were cleaned using the provided III-F filter, and then DNA was captured in a silica resin column to allow washing. Finally, DNA was eluted with DNA/RNase-free water warmed to 37°C prior to elution. Four test DNA samples were analyzed with NanoDrop 2000 (Thermo Fisher) and run on 1% agarose gel (Thermo Fisher) in 1× TAE buffer (40 mM Tris-HCl, 20 mM glacial acetic acid, and 1 mM EDTA, pH 8.3) to confirm the presence of high molecular weight DNA. These four samples were sent to the AAC Genomics Facility at the University of Guelph for quality control analysis using TapeStation gDNA assay (Agilent).

### DNA sequencing

For the 16S rRNA amplicon sequencing, the hypervariable V3–V4 region was chosen to specifically target bacteria, and the resulting amplicons were sequenced in two runs using V3 chemistry (2 × 300 bp) with the Illumina MiSeq platform (sequencing was performed by the AAC Genomics Facility, University of Guelph).

### Sequencing data analysis

Sequencing reads generated by the Illumina MiSeq platform were demultiplexed, resulting in paired-end FASTQ files (forward and reverse reads). Amplicon sequencing data were processed using the OCToPUS pipeline (36). Sequence alignment and operational taxonomic unit (OTU) classification were performed against the SILVA database (version 132). The produced OTU table was normalized using the spike-in provided as described in reference 37. Alpha diversity was assessed using the Chao1 index (species richness) and the Shannon index (richness and evenness). Beta diversity was evaluated using Jaccard distance (Jclass), reflecting community membership, and Bray–Curtis (BrayC) dissimilarity, accounting for relative abundances. Principal coordinate analysis was used for ordination and visualization.

The differentially abundant OTUs (see “Statistical methods”) were further resolved using the oligotyping approach (38), enabling stratification of OTUs into sequence variants. Representative oligotypes were taxonomically classified using NCBI Nucleotide BLAST against 16S rRNA reference sequences with a 97% similarity threshold. Functional potential of microbial communities was inferred using PICRUSt2 (39), and correlation analyses between microbial taxa and cytokine levels, tumor metrics, and treatment doses were performed using the Rhea package (version 1.6). Hierarchical clustering and dendrogram visualization were generated using the ComplexHeatmap package (version 2.16).

### Statistical methods

Differences in tumor burden between groups were assessed using a two-sided Mann-Whitney *U* test. For microbial alpha diversity (Shannon and Chao indices) and differential OTU abundance analyses, assumptions of normality and homogeneity of variance were assessed using the Shapiro–Wilk and Levene’s tests, respectively. Data meeting parametric assumptions were analyzed by one-way ANOVA followed by Tukey’s *post hoc* test; otherwise, the Kruskal–Wallis test was applied, followed by pairwise Mann-Whitney *U* tests with Bonferroni correction. Differences in overall microbial community composition (beta diversity) were evaluated using PERMANOVA (adonis2 function, vegan package) based on both Jaccard (Jclass) and BrayC dissimilarity matrices. Predicted functional profiles derived from PICRUSt2 were analyzed using STAMP (version 2.1.3 [40]) with one-way ANOVA and Tukey’s *post hoc* test, applying an effect-size threshold of 0.25.

Survival analysis was performed on the data collected by 20 weeks of age, with animals still alive at this time point treated as right-censored observations. Median lifespan was calculated using the Kaplan-Meier estimator with 95% confidence intervals. Overall differences in survival distributions among all groups were assessed using the multivariate log-rank test. Pairwise comparisons between groups were performed using the log-rank test, with *P*-values adjusted for multiple comparisons using the Bonferroni correction method. Hazard ratios with 95% confidence intervals were estimated using Cox proportional hazards regression, with the Control group as the reference category. Survival analyses were conducted using Python with the lifelines package for Kaplan-Meier estimation, log-rank tests, and Cox regression; statsmodels for multiple testing corrections; and matplotlib for visualization. All statistical tests were two-sided with a significance threshold of α = 0.05.

## RESULTS

### Chronic gamma irradiation alters alpha and beta diversity of the GM of *Apc^Min/+^* mice

**Fig 1:**
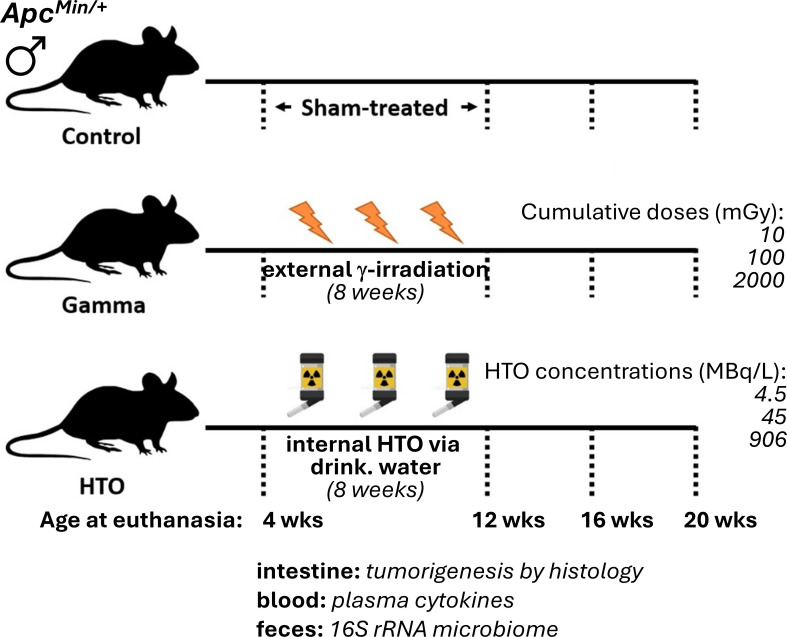
Study design. Male *Apc^Min/+^* mice were treated for 8 weeks continuously with external gamma rays (^60^Co), internal beta radiation (via drinking HTO), or sham-treated. Concentrations of HTO were chosen so that cumulative doses were similar to those in gamma-irradiated animals. Euthanasia and sampling were conducted at the start of exposure (4 weeks of age), upon completion of exposures (12 weeks of age), and 4 and 8 weeks post-exposure (16 and 20 weeks of age, respectively). Six to seven mice per dose per time point were used.

Gamma-irradiated mice were sacrificed upon cessation of exposure, 4 or 8 weeks post-exposure, corresponding to animal ages of 12, 16, and 20 weeks (Fig. 1).

**Fig 2:**
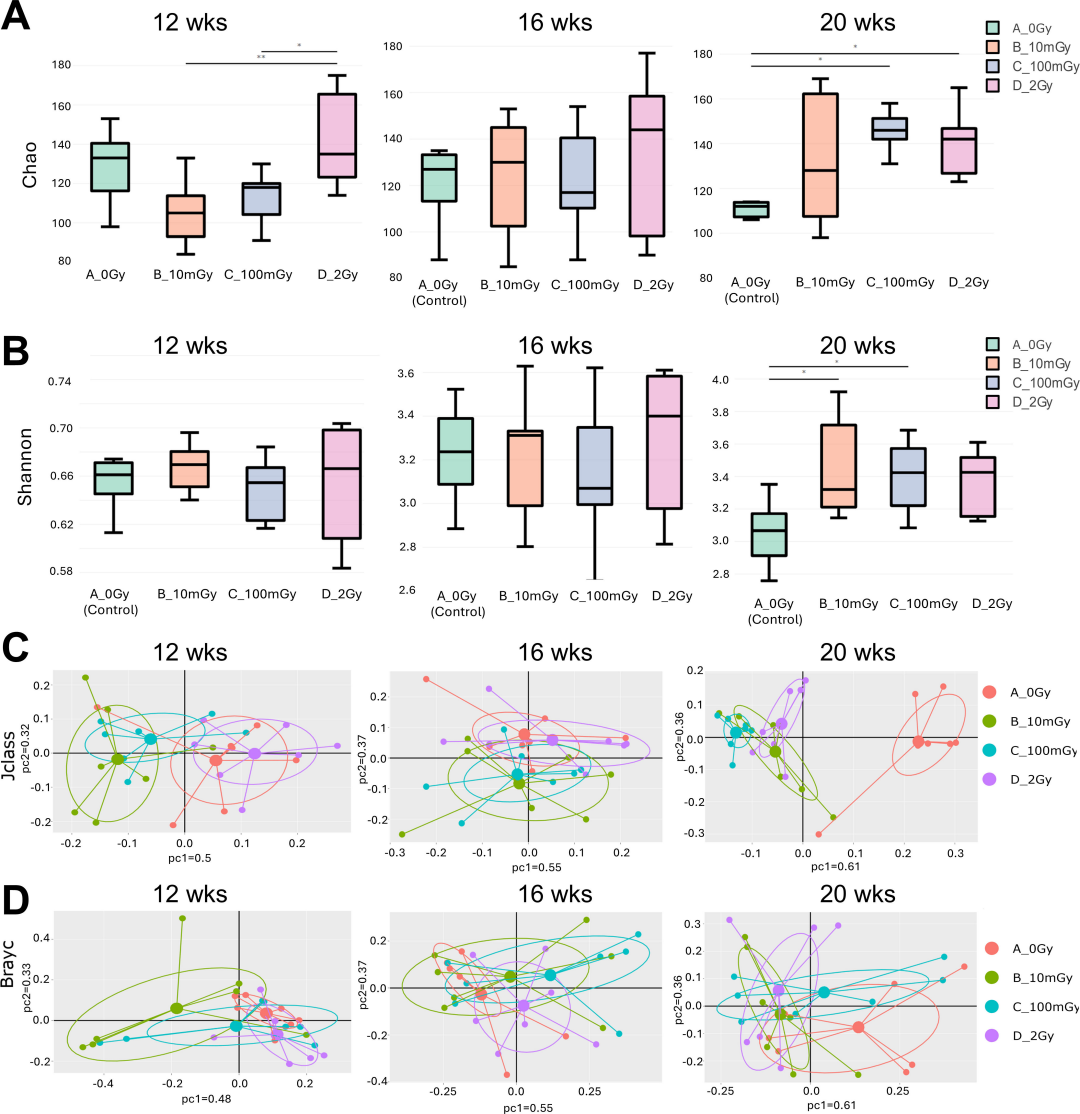
Chronic external gamma irradiation alters microbial diversity within fecal samples of *Apc^Min/+^* mice. For alpha diversity, Chao (**A**) and Shannon (**B**) indices were used to examine bacterial richness and evenness. For beta diversity, principal coordinate analysis of Jaccard distance (Jclass) (**C**) and Bray–Curtis dissimilarity (**D**) of the OTU abundance was used. Statistics were performed using ANOVA (*P ≤ 0.05; **P ≤ 0.01), *n* = 7 per group.

For samples derived from the gamma-exposed mice at 12 weeks of age (immediately upon termination of the 56-day irradiation period), we observed statistically non-significant decreases in the Chao index after low doses of 10 and 100 mGy vs control (Fig. 2A). Interestingly, the 2 Gy exposed group showed a significant difference in the Chao GM diversity compared to the low-dose groups, 10 mGy and 100 mGy. At the same time point, no differences whatsoever were seen using the Shannon index (Fig. 2B). Similarly, no significant changes were observed at the 16-week time point for both indices. At 20 weeks, there was a significant increase in alpha diversity in the 100 mGy and 2 Gy groups compared to the control group using the Chao index (Fig. 2A), and a significant increase for the 10 mGy and 100 mGy groups compared to the control group using the Shannon index (Fig. 2B).

Jclass and BrayC dissimilarity matrices calculated for the gamma-irradiated groups were visualized using principal coordinate analysis and are shown in Fig. 2C and D. At the 12-week time point, there was a significant difference between the 2 Gy group and the 10 mGy (*P* ≤ 0.001) and 100 mGy (*P* ≤ 0.01) groups, using the Jclass method, confirming the Chao differences between the high and low doses at this time point (Fig. 2C). No significant differences were seen at the 16-week time point using both methods, as also observed by alpha diversity. Significant differences were observed at the 20-week time point: using Jclass, significant differences were seen between the control and the 10 mGy, 100 mGy, and 2 Gy groups (all values *P* ≤ 0.001). There were also significant differences between the 2 Gy and the 10 mGy (*P* ≤ 0.01) and 100 mGy (*P* ≤ 0.001) groups. Using the BrayC method for the 20-week time point, significance was only observed between the control and 2 Gy groups (*P* ≤ 0.01; Fig. 2D).

Overall, no significant gamma irradiation effects were observed either immediately or 4 weeks after irradiation, with the exception of an immediate response at the 2 Gy dose. However, notable microbial shifts were detected 8 weeks post-irradiation, indicating a delayed impact on both alpha and beta diversity. Notably, patterns of responses to low doses vs high doses were somewhat distinct.

### Chronic exposure to HTO alters alpha and beta diversity of the GM of *Apc^Min/+^* mice

The gamma irradiation experiment represented a model of external chronic exposure, serving as a reference for qualitatively relating the effects of internal beta radiation from ingested HTO. This is made possible by the use of the HTO concentrations in drinking water that result in cumulative absorbed doses similar to those used in the gamma irradiation experiment. Subsequently, a parallel study was conducted where *Apc^Min/+^* mice were given HTO in drinking water for the same period of time (Fig. 1). Thus, similar to the gamma study, the alpha and beta diversity were examined within fecal samples from HTO-exposed mice.

**Fig 3:**
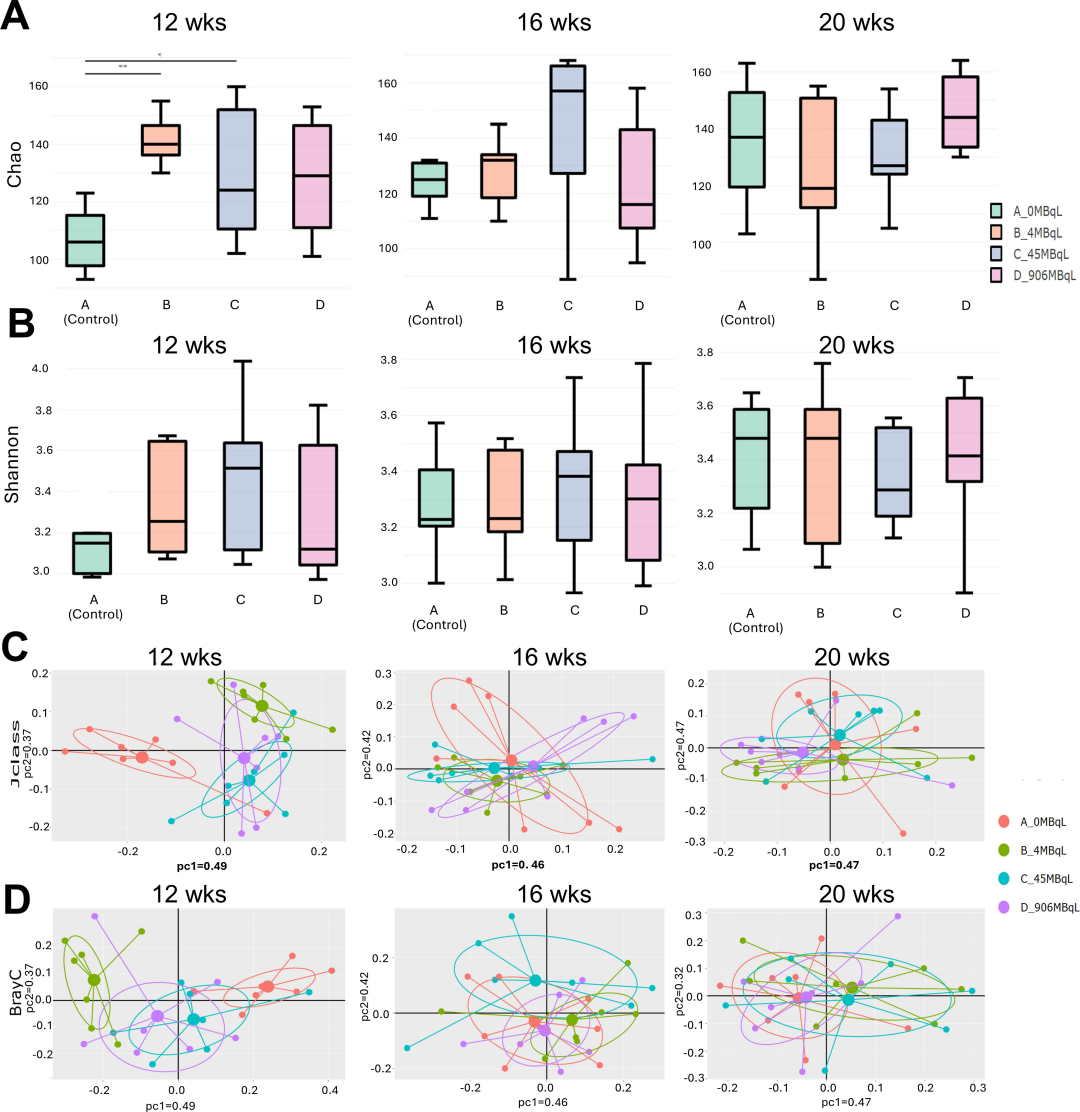
Chronic internal beta-irradiation from ingested HTO alters microbial diversity within fecal samples of *Apc^Min/+^* mice. For alpha diversity, Chao (**A**) and Shannon (**B**) indices were used to examine bacterial richness and evenness. For beta diversity, principal coordinate analysis of Jaccard distance (Jclass; **C**) and Bray–Curtis dissimilarity (**D**) of the OTU abundance was used. Statistics were performed using ANOVA (*P ≤ 0.05; **P ≤ 0.01), *n* = 7 per group.

The Chao diversity index revealed significant differences in species richness at week 12 between the control (designated on plots as A_0MBqL, referring to the concentration of HTO used in drinking water) and both 10 mGy (B_4MBqL) and 100 mGy (C_45MBqL) doses (*P* ≤ 0.01; Fig. 3A). However, Shannon results showed no significant differences for 12 weeks, and no significant changes in alpha diversity were observed at later time points regardless of the index (Fig. 3A and B). At the 12-week time point using the Jclass method, there was a significant difference between the control and the 10 mGy (*P* ≤ 0.001), 100 mGy (*P* ≤ 0.01), and 2 Gy doses (*P* ≤ 0.01; Fig. 3C). Using the BrayC method, there were significant differences between the control and 10 mGy (*P* ≤ 0.001) and 2 Gy (*P* ≤ 0.001) doses, and a significant difference between the low doses, 10 mGy vs 100 mGy (*P* ≤ 0.001) at the 12-week time point (Fig. 3D). At the 16-week time point, there was a significant difference between the 10 mGy and 100 mGy doses (*P* ≤ 0.001), observed in the Jclass method only (Fig. 3C). No significant differences were observed for the fecal samples derived from HTO-treated mice at the 20-week time point. This could indicate that the effect of HTO exposure at low doses is more prominent on species present in lower abundance at the end of exposure, and that the microbiome is restored at later time points using both alpha and beta indices. This could indicate that the effect of low-dose radiation is more prominent on species present in low abundance.

### Cladograms show changes in bacterial speciation in the GM of gamma-irradiated *Apc^Min/+^* mice

To identify taxonomic groups that differed across time points, we performed statistical differential abundance testing. This approach identifies specific features (e.g., organisms, clades, and OTUs) that are most likely to explain observed differences between microbiome populations. The analysis focuses on features that show statistically significant variation between biological classes.

**Fig 4:**
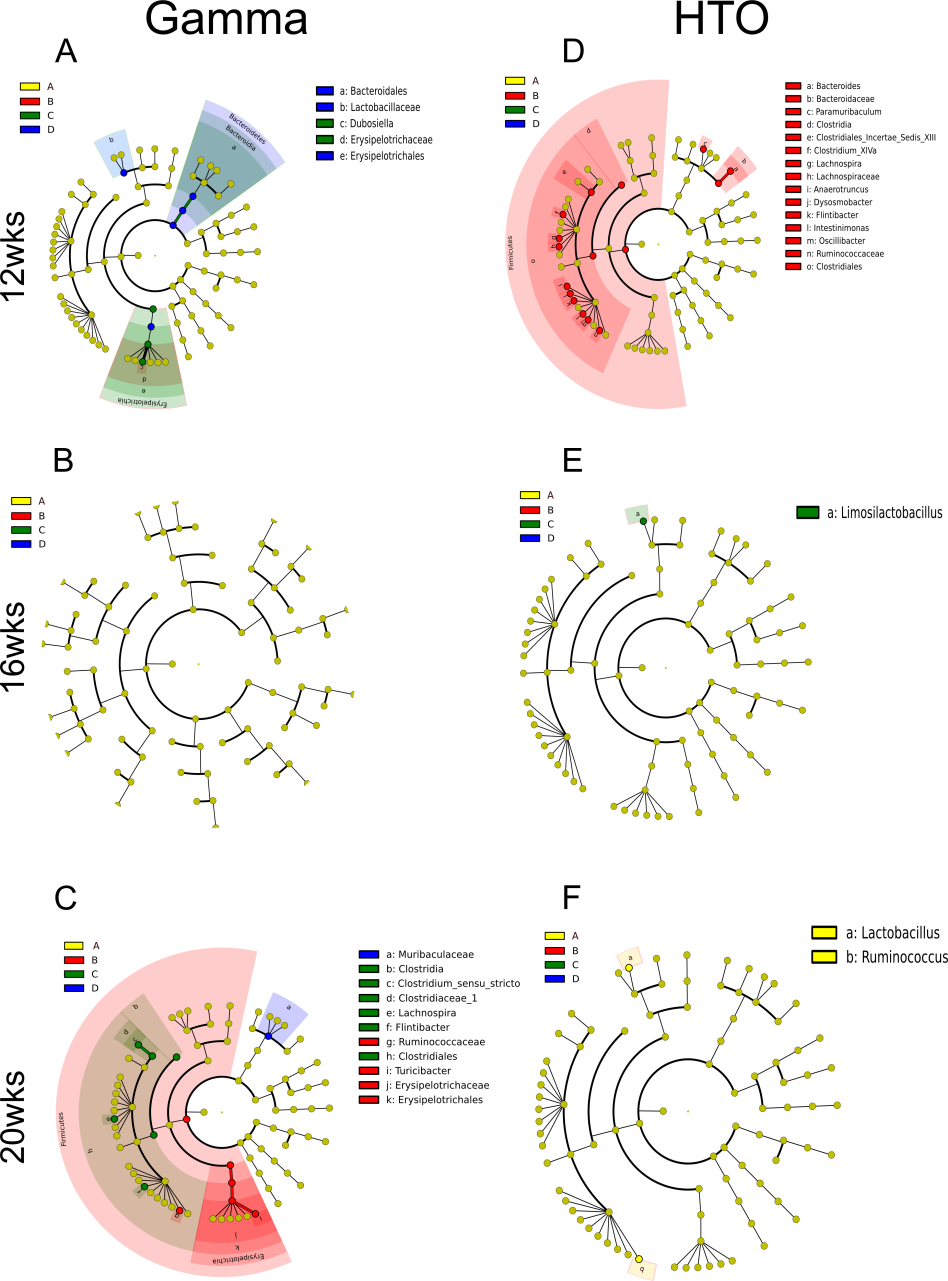
Cladograms showing differentially abundant bacterial taxa in fecal samples from *Apc^Min/+^* mice exposed to gamma radiation (**A–C**) or HTO (**D–F**). Differential abundance was assessed at various time points. Groups A, B, C, and D shown on the legends of the cladograms correspond to cumulative doses of 0 mGy (control), 10 mGy, 100 mGy, and 2 Gy, respectively.

Results of the differential abundance analysis for fecal samples from gamma-irradiated *Apc^Min/+^* mice are shown as cladograms in Fig. 4A through C. At the 12-week time point, the 100 mGy dose was associated with significant changes in taxa belonging to the genus *Dubosiella* and the order Erysipelotrichales (Fig. 4A). Additionally, at this time point, the 2 Gy dose resulted in significant alterations in the class Erysipelotrichia, the order Bacteroidales, and the family Lactobacillaceae. At 20 weeks of age, the 10 mGy dose significantly affected the relative abundance of the genus *Turicibacter*, families Ruminococcaceae and Erysipelotrichaceae, as well as the order Erysipelotrichales. For the 100 mGy dose, significant changes were detected in members of the class Clostridia, order Clostridiales, family Clostridiaceae_1, and multiple genera, including *Clostridium sensu stricto*, *Lachnospira*, and *Flintibacter*. In contrast, the 2 Gy dose at this time point induced a significant shift only in the family Muribaculaceae (Fig. 4C). Together, these findings indicate that chronic gamma irradiation induces dose- and time-dependent shifts in specific bacterial taxa, with particularly pronounced alterations in members of the Erysipelotrichales, Clostridia, and Ruminococcaceae lineages at later stages.

### Cladograms show changes in bacterial speciation in the GM of HTO-treated *Apc^Min/+^* mice

A similar differential abundance analysis was performed on the 16S rRNA sequencing data from fecal samples of HTO-treated mice. At the 12-week time point, the most prominent changes were observed in the 4.5 MBq/L group (corresponding to a 10 mGy dose), where affected taxa included members of the genus *Bacteroides* within the family Bacteroidaceae, as well as the genus *Paramuribaculum* (Fig. 4D). At 16 weeks, the 45.3 MBq/L HTO (corresponding to a 100 mGy dose) resulted in a significant change limited to the genus *Limosilactobacillus* (Fig. 4E). At 20 weeks, the control group had a significantly altered abundance of the genera *Lactobacillus* and *Ruminococcus* (Fig. 4F), indicating a change from the irradiated groups. Overall, these results suggest that internal HTO beta-irradiation primarily perturbs Bacteroidota-associated taxa at early time points, followed by more targeted changes in *Lactobacillus* and *Ruminococcus* at later stages, consistent with a distinct pattern of microbiome remodeling compared with gamma irradiation.

### Predicted microbial functional shifts differ by radiation quality, dose, and time

To assess whether the observed taxonomic shifts were accompanied by changes in predicted functional potential, we inferred microbial pathways using PICRUSt2 and compared pathway abundances within each irradiation cohort at each time point (Materials and Methods). Predicted functional profiles differed by radiation quality, dose, and age, revealing distinct temporal patterns in HTO- vs gamma-exposed mice (Fig. S1). In the HTO exposure cohorts, biosynthesis of ansamycins was enriched at 12 weeks (most pronounced in the 4.5 MBq/L, corresponding to 10 mGy cumulative dose), whereas nitrotoluene degradation was most prominent at 16 weeks (control and 906 MBq/L groups, the latter corresponding to 2 Gy dose). By 20 weeks, several metabolic pathways, including carbon fixation in photosynthetic organisms, D-alanine metabolism, and protein export, were enriched across most dose groups (Fig. S1). In the gamma-irradiated cohorts, biosynthesis of ansamycins was also enriched at 12 weeks (control and 10 mGy), while 16-week samples showed enrichment of penicillin and cephalosporin biosynthesis in the 10 mGy group and activation of the calcium signaling pathway in the 100 mGy group. At 20 weeks, glycan degradation emerged as the most enriched functional signature, particularly across the control and 100 mGy groups (Fig. S1).

### Intestinal tumorigenesis and survival of irradiated *Apc^Min/+^* mice

**Fig 5:**
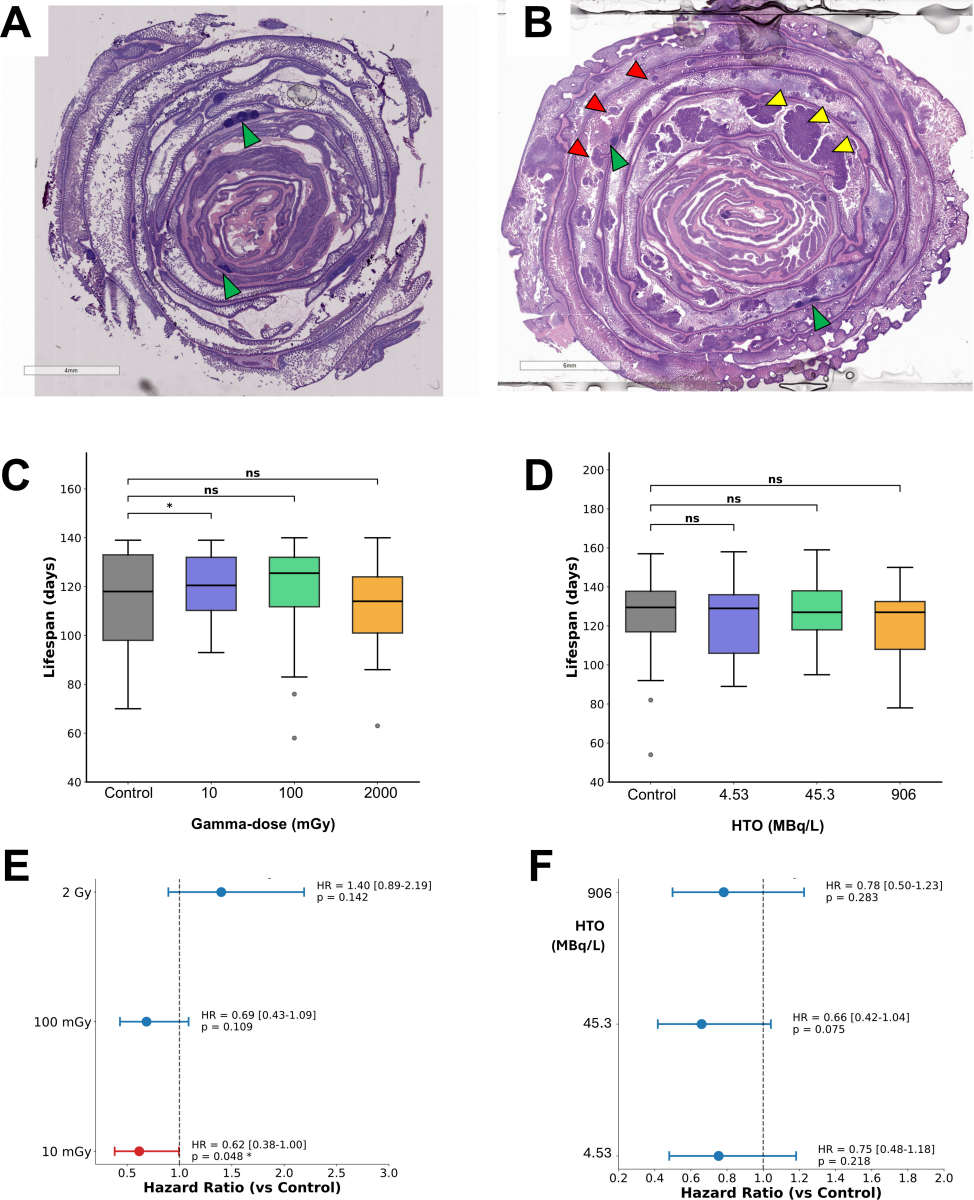
Intestinal pathology and survival outcomes in *Apc^Min/+^* mice following gamma- and internal beta radiation exposures. (**A**) Representative H&E-stained Swiss roll preparation of the intestinal tract from a wild-type C57BL/6 mouse at 16 weeks of age, demonstrating normal mucosal architecture. Green arrows indicate Peyer’s patches. (**B**) H&E-stained Swiss roll preparation from an *Apc^Min/+^* mouse at 16 weeks of age, showing characteristic adenomatous polyps throughout the intestinal epithelium. Yellow and red arrows indicate exemplary large and small adenomas, respectively; green arrow indicates Peyer’s patches. (**C and D**) Box and whisker plots depicting lifespan distribution of deceased male *Apc^Min/+^* mice up to 20 weeks of age (study endpoint) post-irradiation for gamma radiation (**C**) and HTO exposure (**D**). Boxes represent the interquartile range (25th–75th percentiles), horizontal lines indicate the median, and whiskers extend to minimum and maximum values. Significance brackets indicate pairwise comparisons vs control using the log-rank test. *P < 0.05; ns, not significant. (**E and F**) Forest plots showing hazard ratios (HRs) with 95% confidence intervals derived from Cox proportional hazards regression for gamma (**E**) and HTO (**F**) exposure groups, with control as the reference. HR < 1 indicates reduced mortality risk relative to control; HR > 1 indicates increased mortality risk. Red symbols denote statistically significant differences (*P* < 0.05). Sample sizes: gamma: control (*n* = 41), 10 mGy (*n* = 53), 100 mGy (*n* = 58), and 2 Gy (*n* = 54); HTO: control (*n* = 52), 10 mGy (*n* = 50), 100 mGy (*n* = 50), and 2 Gy (*n* = 51).

To assess tumorigenesis and overall systemic health effects in male *Apc^Min/+^* mice, histopathology of the intestinal tract and mouse survival analyses were carried out. As can be seen from the representative intestinal Swiss roll sections, in wild-type C57BL/6 mice, normal mucosal architecture was preserved (Fig. 5A), whereas *Apc^Min/+^* mice exhibited numerous adenomatous polyps throughout the intestinal epithelium (Fig. 5B). Because progression of adenomatosis significantly influences overall health status, we assessed survival as a supportive endpoint through 20 weeks of age in male *Apc^Min/+^* cohorts exposed to chronic external ^60^Co gamma radiation or internal HTO beta radiation (Fig. 5C through F). In the gamma-irradiated cohort, survival exhibited a non-monotonic pattern, with the 10 and 100 mGy groups trending toward improved outcomes relative to controls, whereas the 2 Gy group trended toward reduced survival (Fig. 5C). Consistent with this pattern, Cox proportional hazards modeling showed reduced mortality hazard in the 10 mGy group relative to control (Fig. 5E), while the 100 mGy group showed a similar (non-significant) trend, and the 2 Gy group showed an increased (non-significant) hazard (Fig. 5E). In contrast, survival did not differ significantly among HTO exposure groups through 20 weeks, and hazard ratios for all HTO doses were below 1.0 but did not reach statistical significance (Fig. 5D and F). Overall, these 20-week survival snapshots suggest a radiation-quality-dependent, non-monotonic trend that is more evident after external γ-irradiation than after internal HTO exposure, providing supportive context for the tumor and microbiome endpoints assessed below.

### Intestinal tumorigenesis in gamma-irradiated *Apc^Min/+^* mice

**Fig 6:**
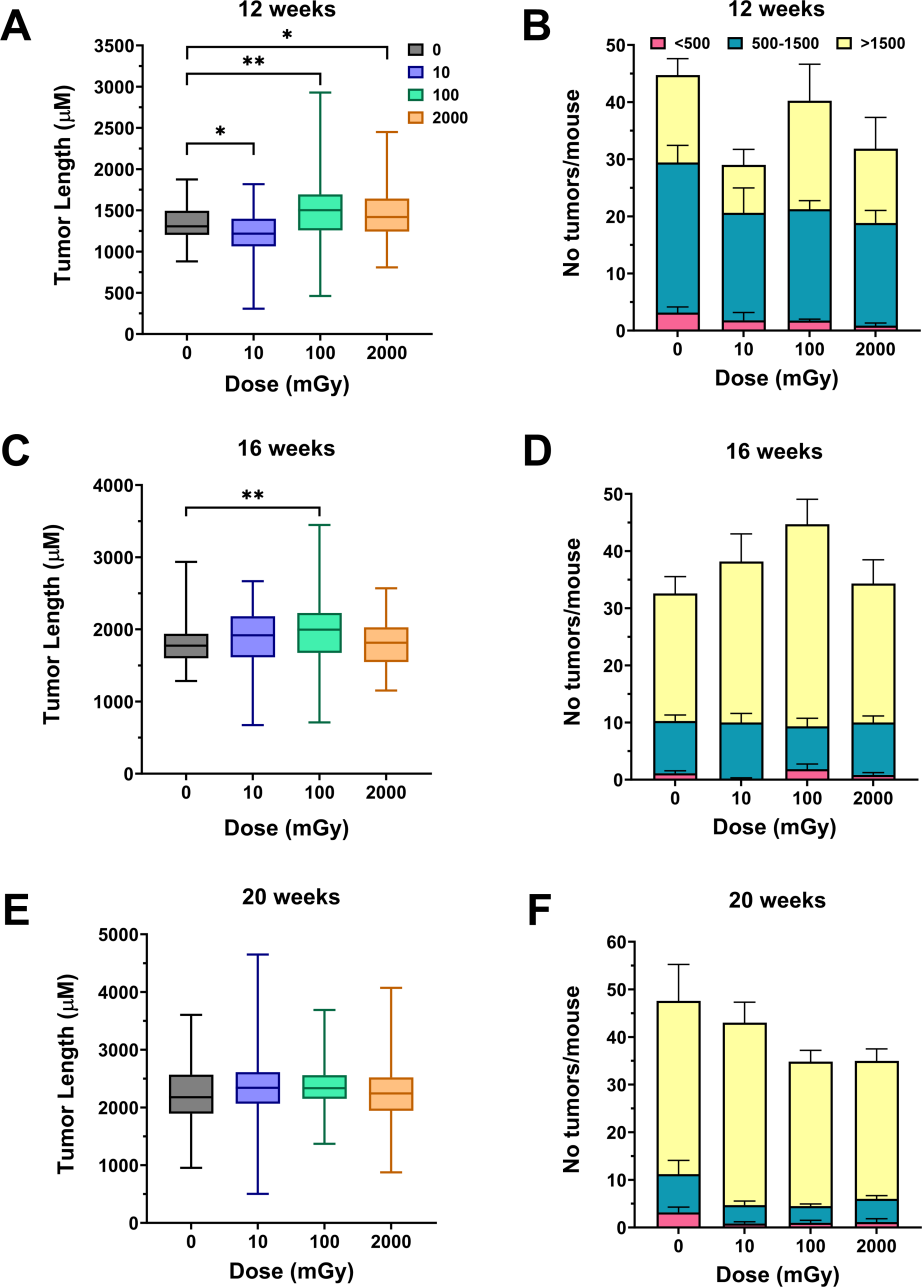
Intestinal tumor lengths and multiplicity in gamma-irradiated *Apc^Min/+^* mice. Tumor lengths (**A, C, and E**) and tumor counts (**B, D, and F**) were assessed using histological sections of intestinal Swiss rolls. For each Swiss roll, 8–16 sections were prepared at intervals of 80–150 µm to ensure that all tumors larger than at least 150 µm were captured and included in the analysis. Statistics were performed using a pairwise Mann-Whitney test (*P < 0.05; **P ≤ 0.01; ****P ≤ 0.0001).

To correlate with the microbiome analysis, intestinal tumorigenesis measurements from the same mice as the fecal samples were taken. This was conducted using histologically processed, intestinal Swiss rolls, with tumors individually counted and measured. It can be seen in Fig. 6A and B that control unirradiated mice by the age of 12 weeks developed about 44 tumors per mouse, with the majority being of intermediate size between 500 and 1,500 µm with a mean length of about 1,300 µm. At this time point, there was a significant decrease in tumor lengths in the 10 mGy vs control, whereas the 100 mGy and 2 Gy groups had a significant increase in tumor lengths compared to control (Fig. 6A). The reduced number of tumors per mouse in the 10 mGy group did not reach significance vs control. At the 16-week time point, there was still a significant increase in tumor lengths for the 100 mGy group only compared to the control (Fig. 6C); however, by 20 weeks, there were no significant changes in tumor lengths across the groups (Fig. 6E). The number of tumors was not altered by irradiation across the groups and time points (Fig. 6B, D, and F).

### Intestinal tumorigenesis in HTO-treated *Apc^Min/+^* mice

**Fig 7:**
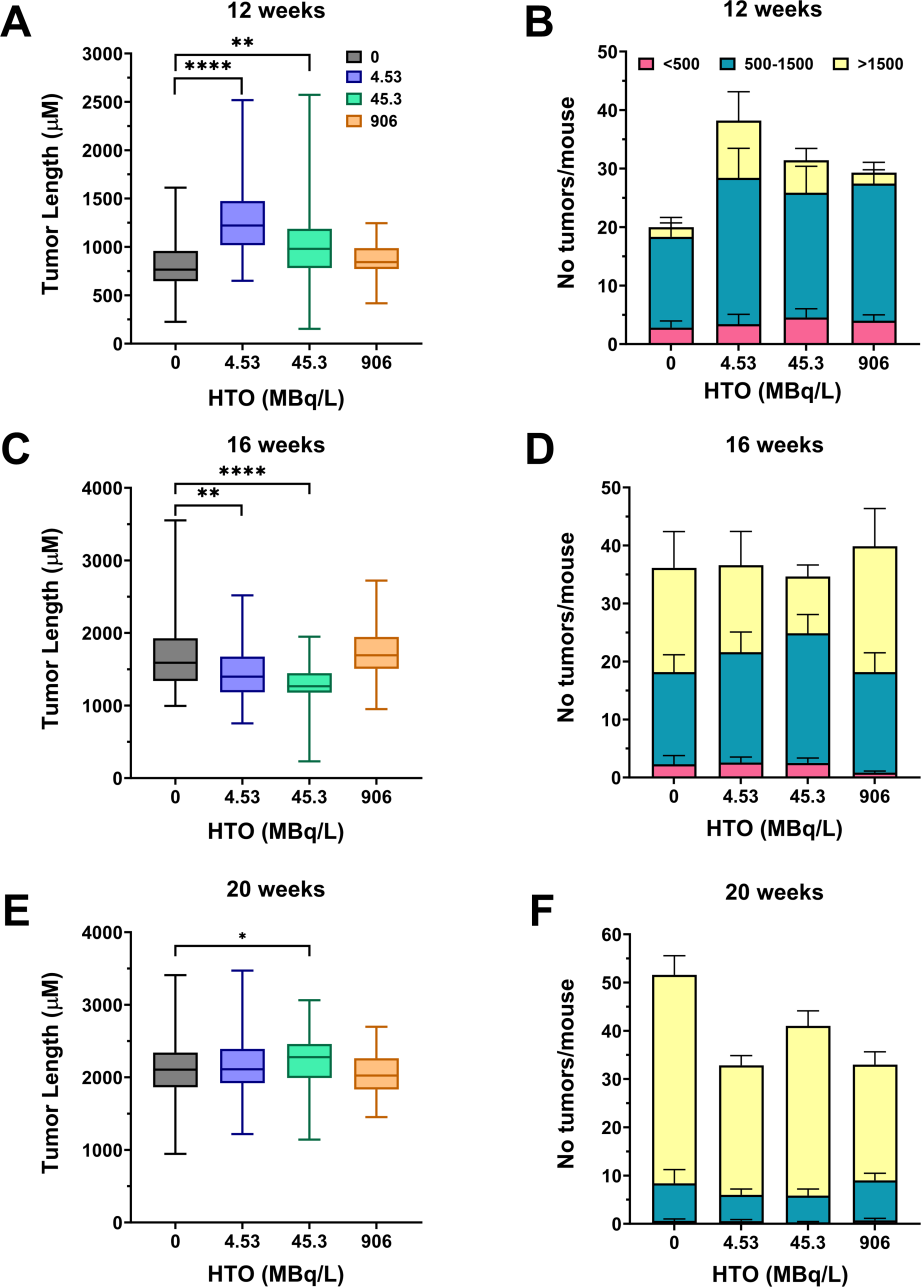
Intestinal tumor lengths and tumor counts in HTO-exposed *Apc^Min/+^* mice. Tumor lengths (**A, C, and E**) and tumor counts (**B, D, and F**) were assessed using histological sections of intestinal Swiss rolls. For each Swiss roll, 8–16 sections were prepared at intervals of 80–150 µm to ensure that all tumors larger than at least 150 µm were captured and included in the analysis. Treatment groups are shown in MBq/L, with 0, 4.53, 4.5, and 906 MBq/L groups corresponding to cumulative doses of 0, 10 mGy, 100 mGy, and 2 Gy, respectively. Statistics were performed using a pairwise Mann-Whitney test (*P < 0.05; **P ≤ 0.01; ****P ≤ 0.0001).

As with the gamma-exposed mice, intestinal tissues were taken from the HTO-treated mice for the measurement of tumorigenesis using histopathological assessment. Results showed that at the 12-week time point, immediately following irradiation, there was a significant increase in tumor lengths for the two low-dose groups of 4.53 MBq/L (cumulative dose of 10 mGy) and 45.3 MBq/L (100 mGy) compared to control, but not for 906 MBq/L (2 Gy) (Fig. 7A). There was also a trend of higher tumor counts for the low-dose exposed mice compared to control, although the results were not significant (Fig. 7B). At 16 weeks, this trend was reversed, in which there was a significant decrease in tumor lengths for the low-dose treatment groups, 10 and 100 mGy, compared to the untreated control (Fig. 7C), and no change in adenoma counts (Fig. 7D). By 20 weeks of age, there was a significant difference in tumor lengths between the 100 mGy and control; however, there were no significant differences in tumor lengths or counts between the highest (2 Gy) and lowest (10 mGy) doses vs control (Fig. 7E and F).

These results suggest that the intestinal tumorigenesis in *Apc^Min/+^* mice responds to internal beta radiation in a distinct manner that differs from that induced by external gamma irradiation, and that this response is complex with respect to both dose dependence and temporal dynamics.

### Correlation between the microbiome and tumorigenesis, dose, and plasma cytokines for gamma radiation

To derive overall biological significance and context of observed changes, we carried out correlation analyses where we assessed how the microbiome OTUs correlate with the rest of the variables, including IR dose. Five tumorigenesis variables were included in this analysis: mean length, tumor count per mouse, the number of small (<500 µm), intermediate (500–1,500 µm), and large adenomas (>1,500 µm) per mouse. We also measured plasma levels of 23 different cytokines using a multiplexed ELISA-based Bio-Plex cytokine kit. Inputting cytokine concentrations from each group into principal component analysis showed high overlap of treatment groups, suggesting limited global changes based on radiation treatment for both gamma (Fig. S2) and HTO experiments (Fig. S3). However, the fact that some individual cytokines were regulated by exposures (e.g., IL-13, MCP-1, G-CSF, and TNF-α for gamma; and MCP-1 and TNF-α for HTO groups [Fig. S3]) in a complex dose- and time-dependent manner suggests the possibility of the activation of specific inflammatory pathways in terms of systemic organismal response. We considered it important to include these cytokine data in the correlation analyses with the microbiota data, as this could help account for a systemic inflammatory status to reveal biologically relevant patterns.

**Fig 8:**
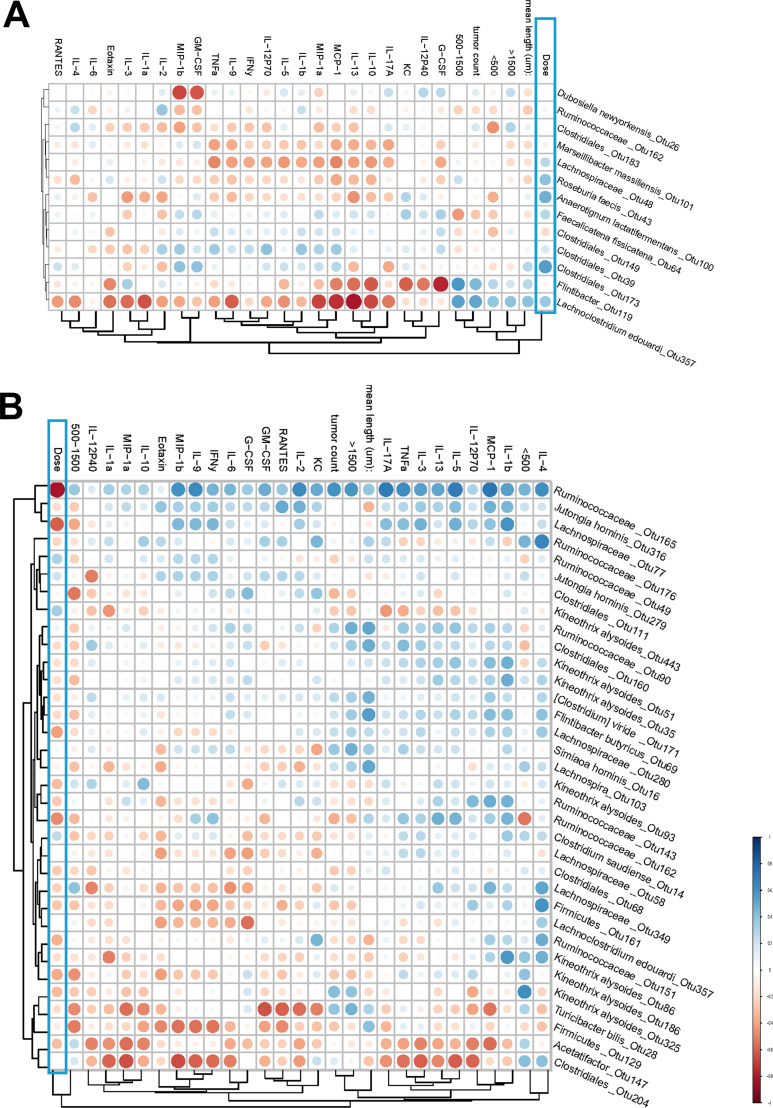
Heatmaps of the correlation matrix between OTUs and non-microbial features for *Apc^Min/+^* mice chronically exposed to gamma radiation. The heatmaps report the hierarchical Ward-linkage clustering based on Pearson’s correlation coefficient among the features. The color depth represents the strength of the correlation, and the size of a circle represents the significance of a respective correlation. Data for 12-week (**A**) and 20-week (**B**) time points are shown. No correlations among the features were found for the 16-week time point.

Figure 8 shows the resulting correlation heatmaps where significantly correlating OTUs are shown vertically, and the non-microbiome variables are clustered in the horizontal direction using the Rhea package. We observed 13 OTUs at 12 weeks and 34 OTUs at 20 weeks, with no correlation found for 16 weeks. This is consistent with the observations of the alpha and beta diversity, where the maximum gamma radiation-induced alterations were seen at 20 weeks and none at 16 weeks (Fig. 2). At 12 weeks, gamma radiation dose clustered together with all five tumor features, separate from the cytokines (Fig. 8A). This cluster correlated positively with OTU-357 *Lachnoclostridium edouardi* of the family Lachnospiraceae. Furthermore, the tumor features, but not dose, correlated positively with the *Flintibacter* genus (OTU-119). The correlations between the cytokines and the OTUs were predominantly negative.

At 20 weeks of age, dose and tumor features were not clustered together anymore (Fig. 8B). Thus, small tumors clustered with IL-4 and were at the maximum distance from dose on the cluster order. The latter grouped with intermediate-sized tumors. The remaining three tumor variables did form a single cluster but were separate from the other two clusters mentioned above. Of note, the majority of microbiome features correlated negatively with dose, with the most pronounced being OTU-165 (Ruminococcaceae sp.) and OTU-77 (Lachnospiraceae sp.). They belonged to a cluster that correlated positively with the vast majority of cytokines, while a cluster of four OTUs showed a negative correlation with the majority of cytokines (OTU-28, OTU-129, OTU-147, and OTU-204).

Overall, these correlation patterns suggest that gamma irradiation-associated microbiome-host relationships are time-dependent, with the dose aligning with tumorigenesis features early (12 weeks) but becoming decoupled by 20 weeks as tumor variables partition into distinct clusters and microbiome OTUs show opposing associations with systemic cytokine signatures.

Taken together, the HTO correlations suggest a more dispersed and time-restricted pattern than observed after gamma irradiation, with weak dose clustering and only isolated OTUs linking dose to tumor burden and inflammatory markers, consistent with distinct radiation-quality-dependent host-microbiome dynamics.

### Correlation between the microbiome and tumorigenesis, dose, and plasma cytokines for HTO-exposed *Apc^Min/+^* mice

**Fig 9:**
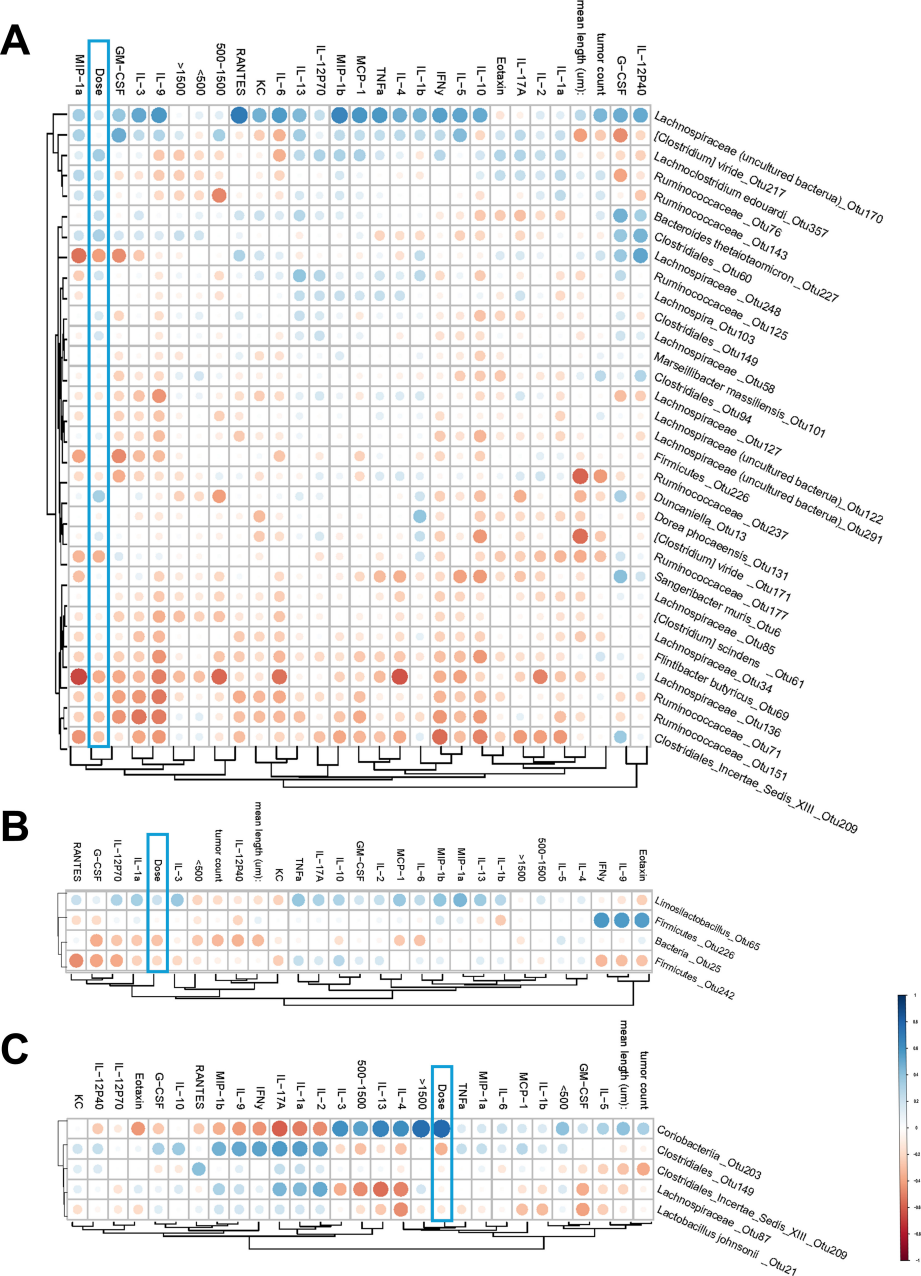
Heatmaps of the correlation matrix between OTUs and non-microbial features for *Apc^Min/+^* mice chronically exposed to internal HTO beta radiation. The heatmaps report the hierarchical Ward-linkage clustering based on Pearson’s correlation coefficient among the features. The color depth represents the strength of the correlation, and the size of a circle represents the significance of a respective correlation. Data for 12-week (**A**), 16-week (**B**), and 20-week (**C**) time points are shown.

Correlation plots for the data obtained in HTO-exposed mice are shown in Fig. 9. It can be seen that 32 OTUs were found to correlate with the non-microbiome features, whereas at 16 and 20 weeks, only 4 and 5 correlating OTUs were identified, respectively. Dose was not clustered well with the five tumor variables at all time points. Except for OTU-203 (Coriobacteriia), which positively correlated with dose for the 20-week time point, no other OTUs demonstrated pronounced correlation with dose at all time points. In addition to dose, OTU-203 strongly correlated positively with large tumors, IL-5, IL-13, intermediate size tumors, and IL-3, and negatively correlated with IL-2, IL-1a, IL17a, interferon-γ (IFN-γ), and Eotaxin (Fig. 9C). Overall, a strong positive correlation with the majority of cytokines was seen for OTU-170 (Lachnospiraceae) at 12 weeks, albeit it correlated weakly with dose and tumor sizes. Altogether, we observed complex patterns of correlation between microbiome OTUs and non-microbiome readouts in *Apc^Min/+^* mice treated with HTO, which were still different from those seen in gamma-irradiated mice.

## DISCUSSION

In this study, we first sought to investigate whether chronic low-dose IR exposure could alter the intestinal microbiome of *Apc^Min/+^* mice, a well-established model of human CRC. We aimed to then correlate the effects of IR on the microbiome with its impacts on intestinal tumorigenesis and systemic inflammatory status to explore potential links between these factors. Additionally, we compared the effects of two radiation qualities, external photon gamma irradiation vs internal beta particle irradiation from ingested tritium, to better understand the relative effects of these radiation types.

**Fig 10:**
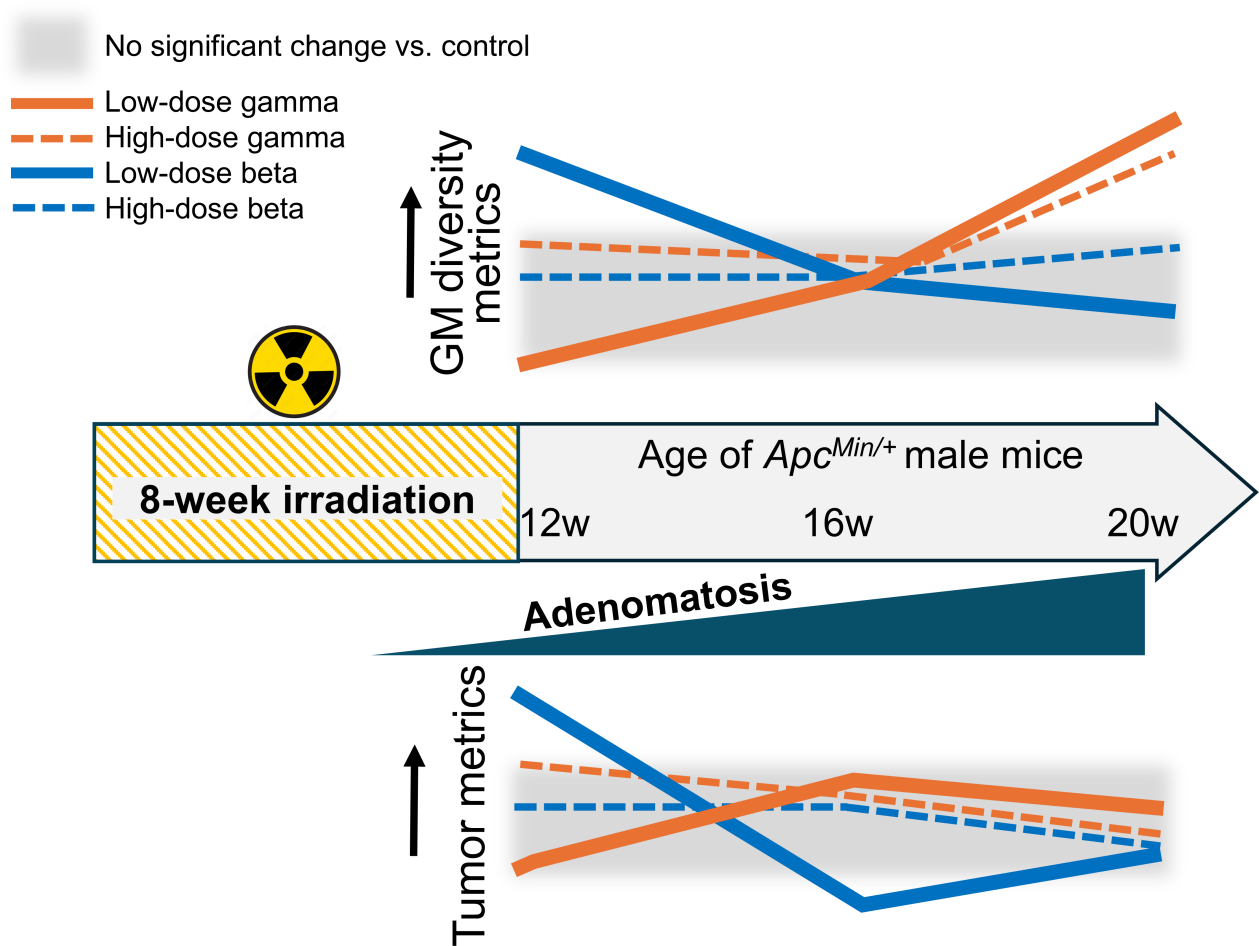
Qualitative summary of the results. The schematic illustrates the dynamics of overall changes in gut microbiome diversity in relation to intestinal tumor metrics in *Apc^Min/+^* male mice chronically exposed to external gamma irradiation vs internal tritium beta-irradiation, as revealed in this study. Distinct trends were observed between the two different radiation types, as well as between low and high doses within each radiation type.

Our findings revealed that both gamma- and HTO beta-irradiation altered the GM diversity, though in distinct and complex ways (qualitatively summarized in Fig. 10). The alpha diversity of the GM in gamma-irradiated mice remained unchanged until 20 weeks of age (8 weeks after the cessation of exposure) when both Shannon and Chao indices were altered by IR (Fig. 2). In a study by Liu et al*.* (41), the authors observed alterations in both alpha and beta diversity of the GM of BALB/C mice exposed to gamma radiation at a cumulative dose of 0.5 Gy, which lasted at least 35 days post-irradiation. While the effects of photon irradiation (gamma- and X-rays)—mostly at high doses—on intestinal microbial communities are well documented (42), there have been no studies investigating the impact of tritium on the GM in mammals.

In contrast, HTO exposure led to an immediate increase in alpha diversity (Chao index) in low-dose-exposed mice at 12 weeks of age. In addition, both beta diversity measures, Jclass and BrayC, were altered in irradiated samples, with a more pronounced effect in low-dose-exposed groups. These results imply that beta radiation can alter both the richness and abundance of intestinal microbial communities, likely driven by changes in less abundant taxa. Interestingly, at the 12-week time point, gamma-irradiated mice exhibited a reduced, though not statistically significant, Chao metric compared to controls, an effect opposite to that induced by HTO. This finding further highlights that chronic external gamma irradiation and internal tritium beta-irradiation exert differential effects on the GM of *Apc^Min/+^* mice at 12 weeks of age, corresponding to the early stage of intestinal tumorigenesis (Fig. 10). By 16 weeks of age, no changes in GM diversity were observed regardless of radiation quality or dose, suggesting a potential recovery or normalization. However, as the mice aged and disease progression continued, GM diversity in gamma-irradiated mice increased (as indicated by the Shannon index for 10 and 100 mGy, and the Chao metric for 100 mGy and 2 Gy) at 20 weeks of age, compared to unirradiated controls. In contrast, the GM diversity metrics in HTO-treated mice did not show significant changes, further supporting the notion that these two radiation types exert qualitatively different effects on the intestinal microbiome.

To our knowledge, no prior studies have examined the effects of tritium beta radiation on mammalian intestinal microbial communities. The only relevant study, which simulated seawater contamination with low concentrations of tritium (3 or 30 KBq/L), demonstrated a shift in the environmental seawater microbiome through altered microorganism abundance (43). Our previous research found that the steady-state tritium concentration in mouse organs after chronic ingestion of HTO was approximately 50% of the tritium concentration in drinking water and was independent of the input levels (35). It follows that the gut microbes of mice in the present study were exposed to approximately 2,000 kBq/L (in the lowest dose group) in their immediate surroundings, thereby being traversed by ionizing beta tracks at a rate similar to that in the seawater study by Lai et al. (43). This suggests that the observed changes in the gut microbiome of HTO-treated mice may result from a direct radiation effect, rather than being mediated by host response to irradiation.

Given the ongoing debate and controversy surrounding the health effects of low doses of IR (44, 45), we compared the effects of 10–100 mGy doses with those of 2 Gy. As evident from Fig. 4, differences were observed between low and high doses within a single radiation type at 12 weeks, which diminished at later time points. This indicates that the gut microbiome responds qualitatively differently to low vs high doses, underscoring the limitations of extrapolating high-dose effects to low-dose scenarios for health risk assessments and emphasizing the need for studies focused specifically on low-dose exposures (≤100 mGy). We found that at the 12-week time point in the tritium-exposed mice, the 10 mGy dose led to significant changes in the genera *Bacteroides* and *Paramuribaculum*. In gamma-irradiated mice, there were significant changes at 12 weeks of age in the 100 mGy cohort in the genus *Dubosiella*, which belongs to the order Erysipelotrichaels. Additionally, there were significant changes in the 2 Gy cohort in the order Bacteroidales (which includes the genus *Bacteroides*), the family Lactobacillaceae, and the order Erysipelotrichales. At the 20-week time point for the HTO-treated mice, our analysis revealed that the control group differed significantly from the irradiated groups by the genera *Lactobacillus* and *Ruminococcus*. A similar pattern was observed in gamma-irradiated mice, where the 10 mGy group showed significant changes in the family Ruminococcaceae. Notably, a recent study in *Apc^Min/+^* mice reported that chronic stress exacerbated tumorigenesis via suppression of *Lactobacillus* taxa in the GM (46). Although our results suggest a complex dynamic pattern of IR-induced alterations in microbial populations over time, the most distinct and potent effects of internal beta radiation appeared at the early 12-week time point, whereas external gamma radiation induced its most prominent effect at 20 weeks. Low doses of HTO predominantly affected taxa within the phylum Bacteroidota at 12 weeks, while gamma radiation, at both low and high doses, primarily altered taxa within the phylum Firmicutes. Notably, no significant changes were seen at the 16-week time point; however, alterations re-emerged at 20 weeks, involving taxa such as *Turicibacter*, the families Erysipelotrichaceae and Ruminococcaceae, as well as the order Erysipelotrichales. Consistent with these compositional shifts, PICRUSt2-based functional inference suggested time- and dose-dependent enrichment of pathways linked to secondary metabolite biosynthesis (e.g., ansamycins and β-lactam pathways) and glycan processing (Fig. S1), supporting radiation-quality-specific modulation of microbial metabolic activity and host-microbiota interactions.

These findings are consistent with previously published work. Using male BALB/c mice exposed to a total absorbed dose of 500 mGy gamma rays (from a ^60^Co source), significant changes in the genus *Bacteroides* were observed, with levels decreasing in a time-dependent manner following radiation exposure (41). Similarly, decreased *Bacteroides* populations were reported in Göttingen minipigs following total body exposure to 1.8 Gy from a linear accelerator photon radiation source. Irradiation also caused a significant increase in *Lactobacillus* and a decrease in *Ruminococcus* genera in the minipigs. In the same study using rhesus macaques, a significant decrease in *Lactobacillus* was observed after 6.3 Gy of photon radiation (47). However, most studies examining the effects of IR on the GM have used high and acute doses of gamma- or X-rays (see the systematic review by Fernandez et al. [21]); therefore, direct comparison with our results is limited.

Two studies that may be more relevant are those investigating wild murine species of bank voles inhabiting Chernobyl territories contaminated with radionuclides. These animals were chronically exposed to both external and internal irradiation at dose rates comparable to those used in our study (27, 48). While the authors reported no significant differences in overall bacterial richness compared to voles from low- or non-contaminated sites, they did observe substantial shifts in abundance disturbance, including an increased Firmicutes/Bacteroidetes ratio. These were the two major phyla affected by both radiation types in our study. A similar shift in the Firmicutes/Bacteroidetes ratio was also observed in the intestinal microbiomes of C57Bl/6J mice exposed to acute X-ray doses of 50–200 mGy, although no changes in diversity or richness were reported (49). Therefore, our results, while consistent with previous studies in one aspect, provide credible new information on the relative effects of chronic low-dose beta radiation from internalized tritium vs external gamma radiation on the GM.

An important strength of our study was the use of the *Apc^Min/+^* mouse model, which provides a highly relevant context for investigating intestinal tumorigenesis. The *Apc^Min/+^* mouse carries a heritable mutation in the *Apc* gene, induced by ethylnitrosourea, which leads to the aggressive development of multiple spontaneous adenomas in the small intestine and, less frequently, in the colon. Given that approximately 85% of human CRC cases are associated with *APC* mutations (50), the *Apc^Min/+^* mouse is widely used to study CRC development and therapeutic interventions. Although the presence of a pre-existing mutation in this model limits its utility for studying the initiation of carcinogenesis following IR, it remains highly relevant for exploring how other factors, including the gut microbiome (51) and environmental conditions (52), influence the promotion and progression of adenomas. This model, therefore, offers a valuable platform for elucidating the interplay between IR-induced effects on intestinal tumorigenesis and IR-driven changes in the gut microbiome.

Our results show that in unirradiated control male *Apc^Min/+^* mice, spontaneous intestinal adenomas appear by 12 weeks and enlarge with age, but their number remains largely stable, which is consistent with the wide range (20–50) reported across facilities and scoring protocols (52–54). Against this baseline, chronic irradiation produced subtle, dose-, time-, and radiation type-dependent modifications rather than a uniform pro-tumorigenic effect. Very low-dose effects were bidirectional: a single 10 mGy gamma exposure transiently reduced adenoma size, whereas the same dose from HTO beta radiation enlarged adenomas at 12 weeks but diminished them by 16 weeks. One hundred milligray of gamma radiation led to an increased tumor size at 12 and 16 weeks of age, and by 20 weeks, both low dose effects were absent. High-dose gamma radiation produced a modest tumor size increase at 12 weeks that dissipated thereafter. Tumor multiplicity was largely unaltered by either radiation quality. Survival assessed through 20 weeks of age was broadly consistent with this non-monotonic pattern for external gamma irradiation (with improved survival metrics at low dose and the opposite trend at 2 Gy), whereas HTO exposure did not significantly alter survival (Fig. 5C through F). Because survival was evaluated as an administratively censored snapshot at 20 weeks and was not a primary focus of this study, these findings should be interpreted cautiously. However, they support the overall nonlinear, transient nature of intestinal tumor responses to low-dose chronic irradiation. As mice age, the influence of both gamma- and beta radiation fades, likely overshadowed by the intrinsic *Apc*-driven adenomatosis. The pattern suggests predominantly nonmutational mechanisms, such as microenvironmental or inflammatory shifts, which are superseded once the *Apc* genetic program fully dictates tumor progression.

Ionizing radiation can induce DNA damage—and, if misrepaired, mutations—that could, in principle, influence tumorigenesis and intersect with host-microbiota dynamics. However, we consider it unlikely that the phenotypes observed here are primarily driven by *de novo* mutational events arising directly from ionization. First, under protracted low dose-rate exposures, much of the damage may remain within repair capacity and therefore may not be efficiently converted into fixed mutations. Second, tumorigenesis in *Apc^Min/+^* mice is dominated by a defined genetic axis, most plausibly loss of the second *Apc* allele, and the probability that direct ionization events specifically target this locus is expected to be low. Instead, if additional mutations do arise in irradiated *Apc^Min/+^* mice, they may be more plausibly linked to secondary, inflammation-associated processes. Given the results of this study, those may include microbiome-mediated modulation of the local intestinal milieu and systemic inflammatory status. Future dedicated studies that profile mutational events across multiple adenomas per animal and integrate these data with longitudinal microbiome and inflammatory readouts will be valuable for testing these mechanistic links.

No previous studies have investigated the effects of environmentally relevant chronic low-dose irradiation on intestinal tumorigenesis in this or related mouse models. The majority of research has focused on acute and/or high doses, primarily using X-rays. For example, Nakayama et al. (55) demonstrated that exposure to 5 Gy X-rays did not increase the total number of adenomas in *Apc^Min/+^* mice, although it significantly enhanced tumor malignancy as measured by invasiveness. Similarly, using X-rays at 2 Gy, Degg et al. (54) reported an approximate 1.8-fold increase in tumor multiplicity in *Apc^Min/+^* ×BALB/c F1 mice. Although both X-rays and gamma rays are forms of photon ionizing radiation, they may elicit different molecular and cellular responses due to their distinct energy characteristics. Current literature suggests that intestinal tumorigenesis in the *Apc^Min/+^* mouse model may be particularly sensitive to these differences. For instance, Kwong et al. (56) observed a 7-fold increase in tumor multiplicity following 5 Gy X-ray exposure, whereas Trani et al. (57) reported only a 1.5-fold increase in the number of intestinal tumors per mouse after a 2 Gy acute gamma radiation dose. Consistent with this trend, a modest 20% increase in the number of intestinal tumors per mouse in *Apc^Min/+^* mice was observed after acute 2 Gy gamma radiation dose in Datta et al. (53), while Ellender et al. (58) found a twofold increase after the same dose of X-rays. Notably, that study also examined a lower dose of 0.5 Gy X-rays, which resulted in a mild 10% increase in adenoma multiplicity, broadly aligning with our findings.

Interestingly, we observed a co-directionality in trends between tumor metrics and GM diversity metrics between 12 and 16 weeks within each radiation type (Fig. 10). Dysbiosis of the GM has been extensively linked to the development and progression of CRC (59, 60), including in the *Apc^Min/+^* mouse model (51). Although increased GM richness is generally associated with protection against colon carcinogenesis (61) and contributes to overall diversity, it is often the specific composition of the microbial community, or particular taxonomic signatures, that defines its impact on colon tumorigenesis (60). This insight suggests that the increased diversity observed in tritium-exposed mice could be associated with larger tumor sizes.

To identify specific taxonomic configurations that may be associated with intestinal adenomatosis in *Apc^Min/+^* mice under irradiation, we conducted correlation analyses between microbiome metrics, represented by OTUs, and physiological metrics, which included five distinct tumor readouts, blood plasma cytokine profiles, and radiation dose. The inclusion of blood cytokine data was based on the understanding that gut dysbiosis and microbial products have been previously linked to chronic inflammation in relation to intestinal adenomatosis (62–64) and metastasis (14). Various bacteria in the microbiome are known to regulate host metabolism and immunity through the secretion of metabolites that can reduce proinflammatory cytokines such as TNF-α, IL-6, and IFN-γ, while promoting the secretion of anti-inflammatory cytokines (25, 65, 66). Notably, similar regulatory mechanisms have been demonstrated in the *Apc^Min/+^* mouse, where inhibition of intestinal adenomatosis was associated with reduced inflammatory cytokines in the blood (67). Moreover, exposure of mice to IR has been shown to induce proinflammatory dysbiosis and intestinal tissue damage, with cytokines playing a key mediating role (68). Given the absence of clear dose-response patterns for tumorigenesis in our study, we incorporated systemic inflammatory markers into our correlation analyses to enhance resolution. This integrative approach may help identify subtle contributors that might be missed using conventional diversity metrics or taxonomic analyses alone.

These analyses revealed several noteworthy observations. For gamma radiation, dose and tumor metrics clustered together at the 12-week time point but not at 20 weeks, suggesting that microbial species identified early after exposure may have played a more prominent role in mediating the effects of gamma radiation on tumorigenesis (Fig. 8A). This pattern was not observed for HTO (Fig. 9A). At 12 weeks, all microbe species positively correlated with gamma dose belonged to the Lachnospiraceae family, whereas both positive and negative correlations were observed for species in HTO-treated mice, also predominantly from the Lachnospiraceae family, with the exception of *Duncaniella* sp. Notably, we identified *Lachnoclostridium edouardi*, which correlated positively and strongly with both dose and all tumor features in gamma-irradiated mice, suggesting a potential role in radiation-induced effect on adenomatosis. *Lachnoclostridium* has been found in higher abundance in patients with ulcerative colitis and inflammatory bowel lesions, indicating its potential as a microbial marker for the non-invasive detection of colorectal tumors and colon cancer (69). Interestingly, the fecal bacteriome of patients with polyps showed a higher abundance of *Lachnoclostridium* compared to CRC patients, suggesting a role for these microorganisms in the early stages of tumorigenesis (70). This finding is consistent with our results, where *Lachnoclostridium edouardi* exhibited a strong correlation with tumor features at 12 weeks, which diminished by 20 weeks. As the disease progresses, the influence of specific microbial taxa may decrease, potentially due to the shifting tumor microenvironment and the evolving immune response. This temporal relationship underscores the importance of early microbiome changes in the context of radiation-induced carcinogenesis.

Notably, *Lachnoclostridium edouardi* also showed a correlation with tumor features and dose in HTO-treated cohorts, despite the distinct patterns observed between gamma and HTO treatments for other parameters. Interestingly, in the HTO-treated mice, it correlated negatively with tumor features and positively with dose. This contrasting behavior may be attributed to the distinct taxonomic profiles in the gut microbiome of gamma-irradiated vs HTO-treated mice, which could shape its overall impact on intestinal adenomatosis.

Other members belonging to the Ruminococcaceae family showed striking positive correlation with tumor features at late stages of tumorigenesis in 20-week-old *Apc^Min/+^* mice irradiated with gamma rays (Fig. 7B). However, it correlated negatively with dose, which is not an easily interpretable finding given the observed lack of the effect of dose on tumor features. In contrast to gamma-irradiated mice, the HTO-exposed mice at 20 weeks did not exhibit significant changes in gut microbiome diversity. Nevertheless, our correlation analysis revealed that Coriobacteriia sp. showed a strong positive correlation with dose, as well as an overall positive correlation with tumor metrics. This class has not previously been implicated in colorectal carcinogenesis, and evidence linking it to other cancers is sparse and controversial. One recent study suggested an inverse association with breast cancer risk (71), while another reported the opposite for hepatocellular carcinoma (72).

Somewhat surprisingly, we did not detect changes in *Fusobacterium nucleatum*, a species strongly associated with adenomatosis in the *Apc^Min/+^* mouse model (73) and human CRC (62). This absence of detection might be due to the use of 16S rRNA sequencing, which, while effective, may lack the sensitivity to identify less abundant taxa like *Fusobacterium nucleatum*. A more sensitive approach, such as shotgun sequencing, which provides better sensitivity for low-abundance species through amplicon sequence variant analysis (74), might reveal the presence of such species. Alternatively, the lack of significant changes may be related to the mild effects of IR on tumorigenesis observed in this study. The chronic irradiation and relatively low doses used may not be sufficient stressors to significantly alter the promotion and progression of intestinal adenomatosis in *Apc^Min/+^* mice, which are known for their aggressive tumor development. To further explore these findings and address the limitation of small intestinal tumor predominance in the *Apc^Min/+^* model, alternative CRC models could be valuable. The novel inducible *KPC:APC* model (75), in which APC (and KRAS) mutations are restricted to the colonic epithelium, would allow investigation of radiation-microbiome interactions specifically in the context of colonic tumorigenesis within an otherwise intact immune environment. Altered interactions between colonic epithelium and gut microbiota preceded adenoma formation in *Apc^Min/+^* mice (51), consistent with observations in a human study that ruled out reverse causation in associating gut microbiome dysbiosis with CRC risk (76). Furthermore, recent findings have shown that colorectal tumors carrying *APC* mutations exhibit distinct microbial and mutational profiles, suggesting that host genetics and the GM may jointly shape tumor evolution (17). This further highlights the relevance of Apc mouse models in studying the impact of IR on the GM and the association of this impact with the CRC risks.

A limitation of this study is that the 20-week time point represents a cohort of mice with advanced intestinal tumors, severe anemia, impaired nutrient absorption, potentially leading to malnutrition, and splenomegaly. Additionally, approximately 40% of *Apc^Min/+^* mice do not survive to this age, making the 20-week-old mice “survivors” nearing end-of-life indicators. In this context, it is possible that the severity of the intestinal cancer masks any subtle, latent IR effects on physiology, contributing to the “normalization” effect observed in the study. Consistent with this possibility, survival at 20 weeks was not detectably worsened even in the 2 Gy group, suggesting that disease progression may dominate overall radiation phenotype at this late stage. Moreover, the most pronounced radiation effects may have been lost with the deceased mice. However, the inclusion of a 16-week time point, where survival is approximately 90% and the trend toward “normalization” was already detectable, provides some reassurance regarding the overall conclusions related to time-dependent dynamics.

An additional limitation relates to the anatomical distribution of tumors in the *Apc^Min/+^* model. While this model predominantly develops adenomas in the small intestine rather than the colon, which differs from the typical presentation of human CRC, several considerations support the translational relevance of our findings. First, the APC mutation driving tumorigenesis in this model is shared with approximately 85% of human colorectal cancers (50). Second, our microbiome analyses were performed on fecal samples, which integrate microbial communities from both the small and large intestine; thus, the observed radiation-induced microbiota changes likely reflect systemic alterations in gut microbial ecology that could influence tumorigenesis throughout the intestinal tract. Third, microbial metabolites and inflammatory signals originating from small intestinal dysbiosis can influence colonic pathophysiology through enterohepatic circulation and systemic immune modulation (65). Nevertheless, future studies employing colon-specific tumor models, such as the inducible *KPC:APC* model (75), would be valuable to directly validate these findings in the context of colonic tumorigenesis.

In conclusion, the results of this study indicate that both external gamma irradiation and internal beta-irradiation from ingested HTO, applied at chronic low-dose rates over an 8-week period, can alter the gut microbial communities in *Apc^Min/+^* mice, as assessed from fecal samples. These alterations were qualitatively different between the two radiation types and did not exhibit a clear dose-dependent pattern. Additionally, the effects of the low cumulative doses of 10 or 100 mGy were somewhat distinct from those of the high dose of 2 Gy for both radiation types. The differences in the impact of gamma and beta radiation on the gut microbiome were consistent with the effects observed on intestinal tumorigenesis, particularly in the overall directionality of time-dependent changes. Taxonomic profile changes were most pronounced at 12 weeks of age, immediately after exposure, for both radiation types. However, gamma radiation primarily affected Firmicutes, whereas HTO treatment predominantly disturbed Bacteroidota taxa. The inclusion of systemic inflammatory profiles, with 23 cytokines measured in blood plasma, in the correlation analyses revealed microbial species that correlated with both dose and tumor metrics. Notably, *Lachnoclostridium edouardi* was found to positively correlate with dose and tumors for both external gamma irradiation and internal beta radiation at the early stages of intestinal adenomatosis. More distinct species profiles were observed in 20-week-old mice, with Ruminococcaceae sp. and Coriobacteriia sp. showing positive correlations with tumorigenesis in HTO- and gamma-treated mice, respectively.

Our findings highlight the importance of considering radiation quality, dose rate, and timing in evaluating the long-term biological impacts of low-dose exposure and suggest that radiation-induced modulation of the gut microbiome may play a mechanistic role in early intestinal and colon tumor promotion, with implications for radiation protection and cancer risk assessment.

## Data Availability

Sequencing data generated in this study have been deposited in NCBI’s SRA archive and are accessible through the BioProject PRJNA1270005 accession number.

## SUPPLEMENTAL MATERIAL

Supplemental Figures msystems.01156-25-s0001.pdf Figures S1 to S3.

ASM does not own the copyrights to Supplemental Material that may be linked to, or accessed through, an article. The authors have granted ASM a non-exclusive, world-wide license to publish the Supplemental Material files. Please contact the corresponding author directly for reuse.
