# Supplementary material for: Impact of chronic low-dose external gamma- and internal tritium beta-irradiation on the gut microbiome in the context of intestinal tumorigenesis in ApcMin/+ mice
Source: mSystems. 2026 Mar 17;11(4):e01156-25. doi: 10.1128/msystems.01156-25 (PMC13098236; doi:10.1128/msystems.01156-25)
Supplement: Supplemental Figures — Figures S1 to S3. [file msystems.01156-25-s0001.pdf]

Supplemental Material for the manuscript

"Impact of chronic low-dose external gamma- and internal tritium beta-irradiation on gut microbiome in the context of intestinal tumorigenesis in *Apc*$^{Min/+}$ mice"

Holly Laakso[1], Manar Hashem Taha[2], Matthew Flegal[1], Joel Surette[1], Mohamed Mysara[2,3]*, Dmitry Klokov[4,5]*

[1] Isotopes, Radiobiology, and Environment Directorate, Canadian Nuclear Laboratories, Chalk River, ON, Canada

[2] Bioinformatics group, Center for Informatics Science, School of Information Technology and Computer Science, Nile University, Sheikh Zayed City, Egypt

[3] Microbial Biotechnology Research Unit, Nuclear Medical Applications, Belgian Nuclear Research Centre (SCK CEN), Mol, Belgium

[4] Laboratory of Experimental Radiotoxicology and Radiobiology, The French Authority on Nuclear Safety and Radioprotection (ASNR), Fontenay-aux-Roses, France

[5] Department of Biochemistry, Microbiology and Immunology, University of Ottawa, Ottawa, Canada

* Corresponding authors

E-mail: dmitry.klokov@asnr.fr (DK)

Email: MMaysara@nu.edu.eg (MM)

Short title: C**hronic irradiation and gut microbiome in Apc$^{Min/+}$ mice**

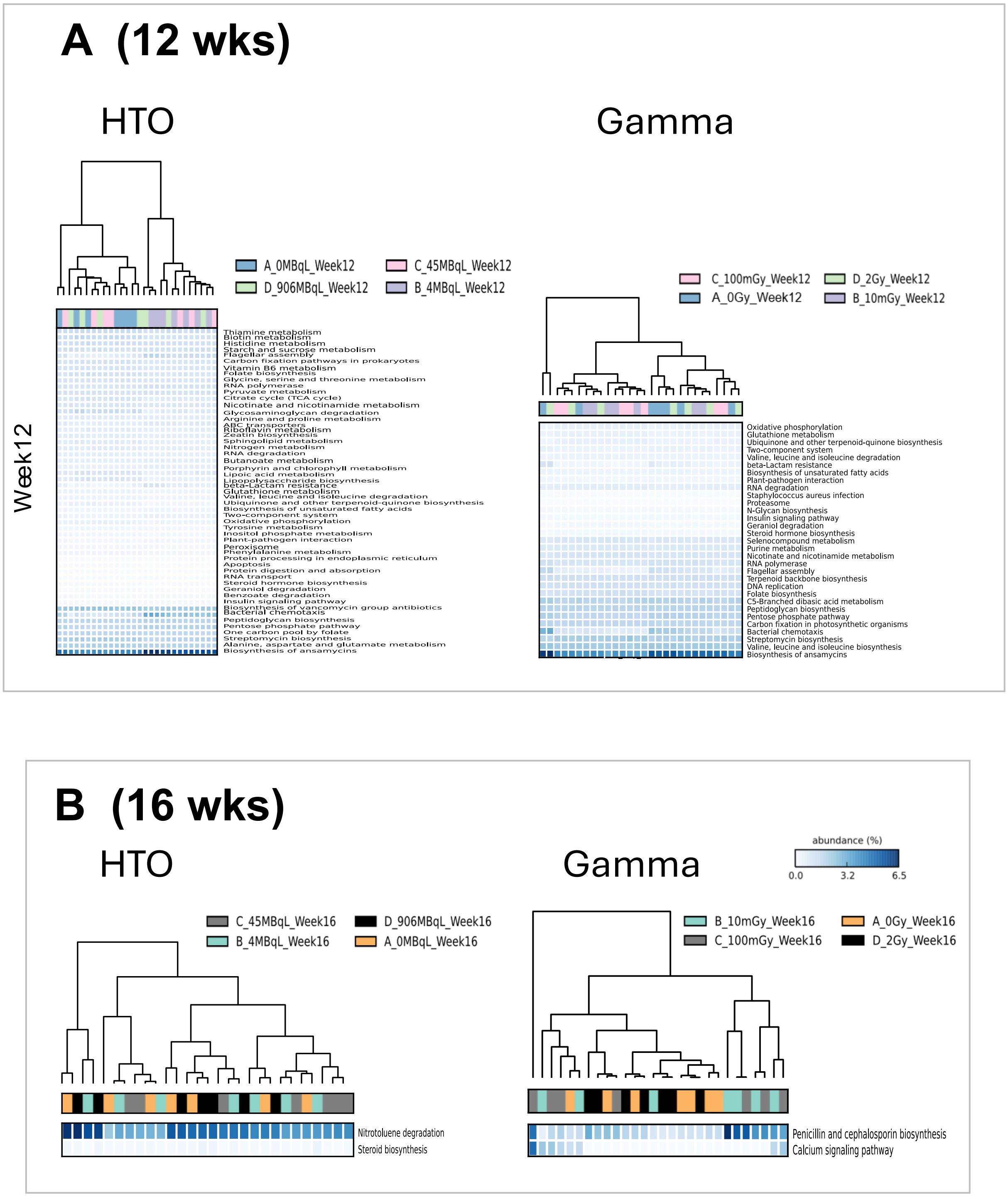

A (12 wks)
HTO
Gamma
A_0MBqL_Week12
C_45MBqL_Week12
D_906MBqL_Week12
B_4MBqL_Week12
Week12
Thiamine metabolism
Biotin metabolism
Histidine metabolism
Starch and sucrose metabolism
Flagellar assembly
Carbon fixation pathways in prokaryotes
Vitamin B6 metabolism
Folate biosynthesis
Glycine, serine and threonine metabolism
RNA polymerase
Pyruvate metabolism
Citrate cycle (TCA cycle)
Nicotinate and nicotinamide metabolism
Glycosaminoglycan degradation
Arginine and proline metabolism
ABC transporters
Riboflavin metabolism
Zeatin biosynthesis
Sphingolipid metabolism
Nitrogen metabolism
RNA degradation
Butanoate metabolism
Porphyrin and chlorophyll metabolism
Lipoic acid metabolism
Lipopolysaccharide biosynthesis
beta-Lactam resistance
Glutathione metabolism
Valine, leucine and isoleucine degradation
Ubiquinone and other terpenoid-quinone biosynthesis
Biosynthesis of unsaturated fatty acids
Two-component system
Oxidative phosphorylation
Tyrosine metabolism
Inositol phosphate metabolism
Plant-pathogen interaction
Peroxisome
Phenylalanine metabolism
Protein processing in endoplasmic reticulum
Apoptosis
Protein digestion and absorption
RNA transport
Steroid hormone biosynthesis
Geraniol degradation
Benzoate degradation
Insulin signaling pathway
Biosynthesis of vancomycin group antibiotics
Bacterial chemotaxis
Peptidoglycan biosynthesis
Pentose phosphate pathway
One carbon pool by folate
Streptomycin biosynthesis
Alanine, aspartate and glutamate metabolism
Biosynthesis of ansamycins
C_100mGy_Week12
D_2Gy_Week12
A_0Gy_Week12
B_10mGy_Week12
Oxidative phosphorylation
Glutathione metabolism
Ubiquinone and other terpenoid-quinone biosynthesis
Two-component system
Valine, leucine and isoleucine degradation
beta-Lactam resistance
Biosynthesis of unsaturated fatty acids
Plant-pathogen interaction
RNA degradation
Staphylococcus aureus infection
Proteasome
N-Glycan biosynthesis
Insulin signaling pathway
Geraniol degradation
Steroid hormone biosynthesis
Selenocompound metabolism
Purine metabolism
Nicotinate and nicotinamide metabolism
RNA polymerase
Flagellar assembly
Terpenoid backbone biosynthesis
DNA replication
Folate biosynthesis
C5-Branched dibasic acid metabolism
Peptidoglycan biosynthesis
Pentose phosphate pathway
Carbon fixation in photosynthetic organisms
Bacterial chemotaxis
Streptomycin biosynthesis
Valine, leucine and isoleucine biosynthesis
Biosynthesis of ansamycins
B (16 wks)
HTO
Gamma
abundance (%)
0.0
3.2
6.5
C_45MBqL_Week16
D_906MBqL_Week16
B_4MBqL_Week16
A_0MBqL_Week16
Nitrotoluene degradation
Steroid biosynthesis
B_10mGy_Week16
A_0Gy_Week16
C_100mGy_Week16
D_2Gy_Week16
Penicillin and cephalosporin biosynthesis
Calcium signaling pathway

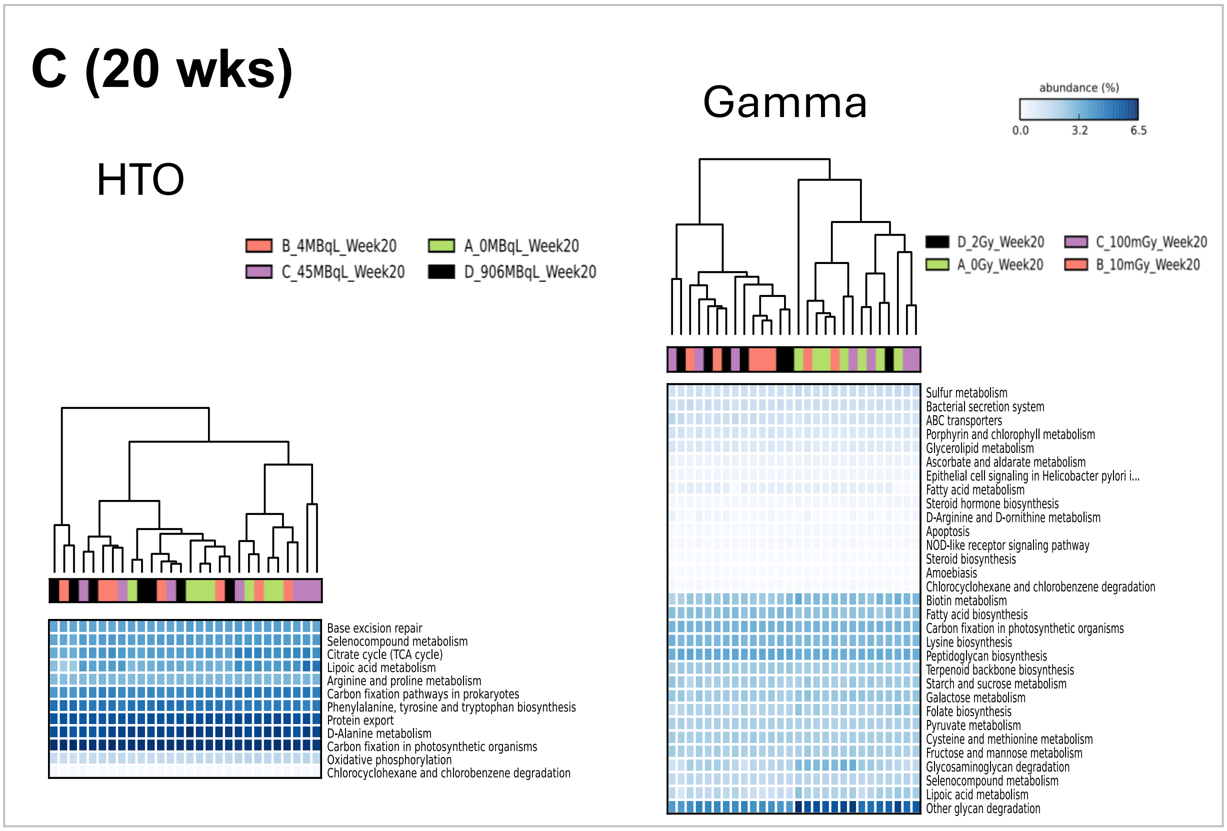

Supplemental Fig S1. PICRUSt2 functional prediction analysis reveals time- and radiation type-dependent alterations in microbial metabolic pathways. Heatmaps with hierarchical clustering depicting predicted functional pathway abundances in the gut microbiome of ApcMin/+ mice following exposure to internal tritiated water (HTO; left panels) or external gamma-radiation (right panels) at various doses. Analyses are shown at **(A)** 12 weeks, **(B)** 16 weeks, and **(C)** 20 weeks post-irradiation.

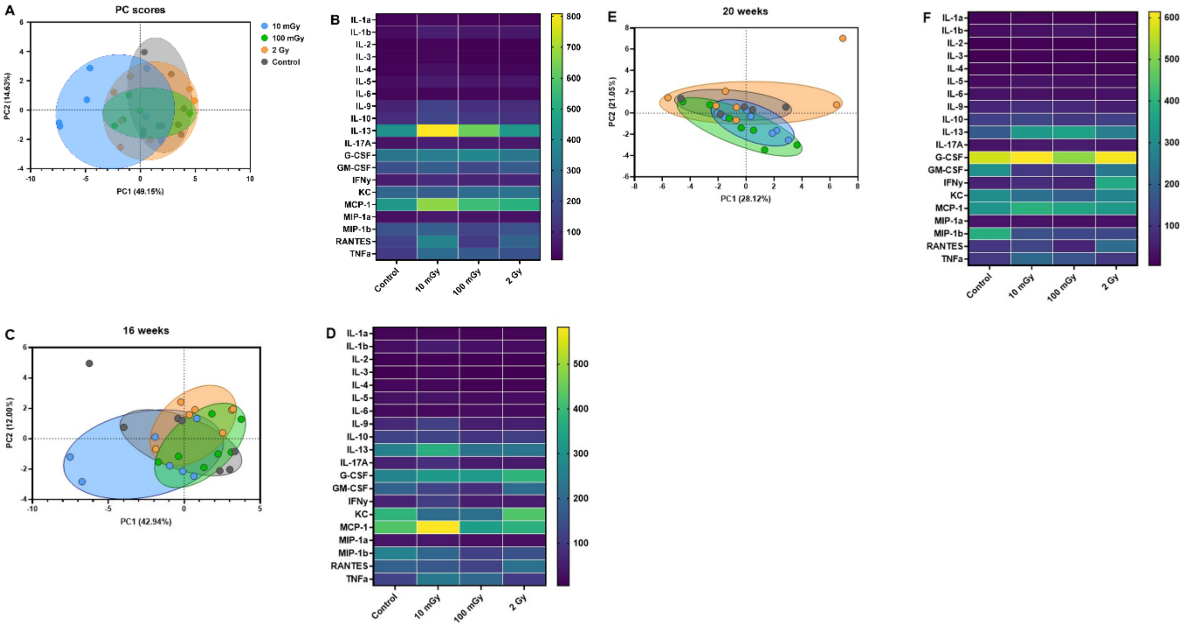

Supplemental Fig S2. Lack of global changes in blood plasma cytokine levels following gamma-irradiation in *Apc*$^{Min/+}$ mice. Principle component analysis of 23 cytokine levels measured in blood plasma of mice exposed to indicated cumulative doses during 8-week irradiation period immediately upon cessation of irradiation **(A)**, 4 weeks **(C)** or 8 weeks **(E)** after irradiation using Bio-Plex high-throughput screening. Heatmaps show mean cytokine concentrations (pg/mL) determined upon cessation of irradiation **(B)**, at 4 weeks **(D)** or 8 weeks **(F)** after irradiation.

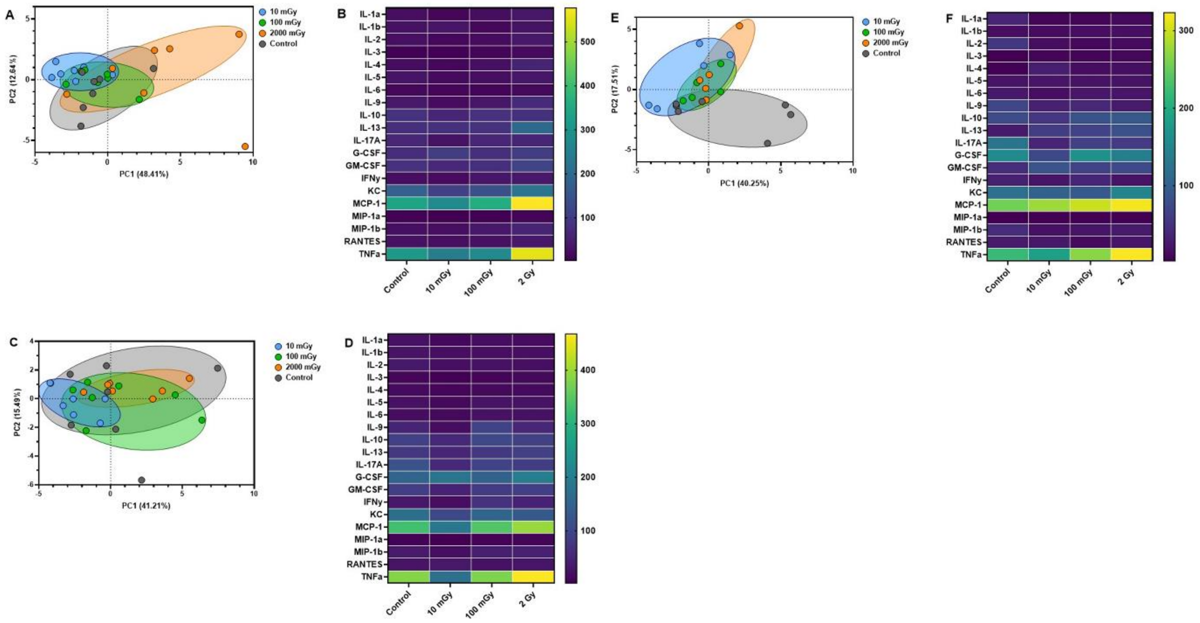

Supplemental Fig S3. Lack of global changes in blood plasma cytokine levels following internal HTO beta-irradiation in *Apc*$^{Min/+}$ mice. Principle component analysis of 23 cytokine levels measured in blood plasma of mice exposed to indicated cumulative doses during 8-week irradiation period immediately upon cessation of irradiation **(A)**, 4 weeks **(C)** or 8 weeks **(E)** after irradiation using Bio-Plex high-throughput screening. Heatmaps show mean cytokine concentrations (pg/mL) determined upon cessation of irradiation **(B)**, at 4 weeks **(D)** or 8 weeks **(F)** after irradiation.
